# Identification of an Intestinal Microbiota Signature Associated With the Severity of Necrotic Enteritis

**DOI:** 10.3389/fmicb.2021.703693

**Published:** 2021-08-19

**Authors:** Qing Yang, Jing Liu, Xiaofan Wang, Kelsy Robinson, Melanie A. Whitmore, Sydney N. Stewart, Jiangchao Zhao, Guolong Zhang

**Affiliations:** ^1^Department of Animal and Food Sciences, Oklahoma State University, Stillwater, OK, United States; ^2^Department of Animal Science, University of Arkansas, Fayetteville, AR, United States

**Keywords:** *Clostridium perfringens*, dysbiosis, necrotic enteritis, microbiota, microbiome, poultry, 16S rRNA gene sequencing

## Abstract

Necrotic enteritis (NE), an economically devastating disease of poultry caused by pathogenic *Clostridium perfringens*, is known to induce small intestinal lesions and dysbiosis. However, the intestinal microbes that are associated with NE severity are yet to be characterized. Here, we investigated the link between the ileal microbiota and disease severity in a chicken model of clinical NE using 16S rRNA gene sequencing. Our results indicated that richness and Shannon Index of the ileal microbiota were drastically reduced (*p*<0.01) as NE was exacerbated. While the relative abundance of *C. perfringens* increased from 0.02% in healthy chickens to 58–70% in chickens with severe infection, a majority of the ileal microbes were markedly diminished, albeit varying in their sensitivity to NE. Compositionally, a large group of ileal microbes showed a significant correlation with NE severity. Firmicutes, such as group A and B *Lactobacillus*, *Lactobacillus reuteri*, *Subdoligranulum variabile*, *Mediterraneibacter*, and *Staphylococcus* as well as two genera of Actinobacteria (*Corynebacterium* and *Kocuria*) and two highly related Cyanobacteria were progressively declined as NE was aggravated. Other Firmicutes, such as *Weissella*, *Romboutsia*, *Kurthia*, *Cuneatibacter*, *Blautia*, and *Aerococcus*, appeared much more sensitive and were rapidly abolished in chickens even with mild NE. On the other hand, *Enterococcus cecorum* and two *Escherichia*/*Shigella* species were only enriched in the ileal microbiota of chickens with extremely severe NE, while several other species such as *Streptococcus gallolyticus* and *Bacteroides fragilis* remained unaltered by NE. Functionally, secondary bile acid biosynthesis was predicted to be suppressed by NE, while biosynthesis of aromatic and branched-amino acids and metabolism of a majority of amino acids were predicted to be enhanced in the ileum of NE-afflicted chickens. These intestinal microbes showing a strong correlation with NE severity may provide important leads for the development of novel diagnostic or therapeutic approaches to NE and possibly other enteric diseases.

## Introduction

Necrotic enteritis (NE), caused by *Clostridium perfringens*, is one of the most economically significant enteric diseases in poultry, resulting in an annual loss of approximately $6 billion to the global poultry industry (Wade and Keyburn, 2015). NE is manifested by lesions throughout the small intestine and associated with growth retardation, reduced feed efficiency, and up to 50% mortality (Shojadoost et al., 2012; Wade and Keyburn, 2015). NE is a multifactorial disease, and a coccidial infection is an important predisposing factor by damaging intestinal epithelial integrity and thus facilitating *C. perfringens* colonization and translocation (Shojadoost et al., 2012; Moore, 2016). *Eimeria*, therefore, is often administered prior to *C. perfringens* infection to induce NE experimentally (Shojadoost et al., 2012; Moore, 2016).

The gastrointestinal (GI) tracts of humans and animals are colonized by trillions of diverse microorganisms that collectively play a critical role in maintaining host health by providing nutrients and energy, modulating immune responses, and working through the competitive exclusion of pathogens (Sun and Jia, 2018; Durack and Lynch, 2019). Dysbiosis or imbalance of the intestinal microbiota has been linked to a variety of enteric diseases such as inflammatory bowel diseases (IBD) in humans (Sun and Jia, 2018; Durack and Lynch, 2019). Similarly, NE has been shown to modify the intestinal microbiota with major perturbations observed in lactic acid-producing *Lactobacillus* and butyrate-producing *Ruminococcaceae* and *Lachnospiraceae* families of bacteria in chickens (Antonissen et al., 2016). However, the results have been inconsistent and different bacteria were altered in different studies. For example, in the ileum or cecum of NE-infected chickens, lactobacilli were reduced in some studies (Zhang et al., 2018; Hernandez-Patlan et al., 2019; Yang et al., 2019), but enriched (Lin et al., 2017; Latorre et al., 2018; Xu et al., 2018) or unaltered (Bortoluzzi et al., 2019; Kiu et al., 2019; Lu et al., 2020) in others. In one study, *Lactobacillus crispatus* increased, while *Lactobacillus johnsonii* was reduced in the cecum of chicken in response to NE (Stanley et al., 2012). Moreover, different clostridia, well beyond *Ruminococcaceae* and *Lachnospiraceae*, were differentially regulated by NE in different studies (Stanley et al., 2012; Latorre et al., 2018; Lu et al., 2020). Notably, varying severity degrees of NE were induced in these studies, and the link between intestinal microbiota and NE severity has not been well-elucidated. Therefore, there is a need to analyze the chicken microbiota separately according to their disease severities and more importantly, examine whether and how the intestinal microbiota are influenced by the severity of NE.

In this study, we employed a chicken model of clinical NE through a coinfection of broilers with *Eimeria* and *C. perfringens*. Animals displayed a broad range of clinical symptoms with a varying degree of lesions in the small intestine. Chickens were grouped based on the severity of the intestinal lesions, and the correlation between their ileal microbiota profiles and disease severity was examined. We observed a drastic alteration in the microbiota composition and functional potential. The most dominant bacteria were shifted from *Lactobacillus* to *C. perfringens* in the ileum, concomitant with a dramatic reduction in richness. Most bacteria were increasingly diminished or abolished as NE became more severe, but with different patterns in the changes in abundance. A number of bacteria were strongly correlated with NE severity. Identification of the intestinal microbiota signature for NE severity may help with disease diagnosis and provide important leads for the control and prevention of NE without relying on antibiotics.

## Materials and Methods

### Chicken Co-infection Model of Clinical NE

All animal procedures described below have been approved by the Institutional Animal Care and Use Committee (IACUC) of Oklahoma State University under the protocol number AG-16-10. A chicken model of clinical NE was employed through co-infection of *Eimeria maxima* and *C. perfringens* as previously described with slight modifications (Cooper and Songer, 2010; Shojadoost et al., 2012). Briefly, day-of-hatch unvaccinated male Cobb broiler chicks were obtained from a Cobb-Vantress Hatchery (Siloam Springs, AR, United States), tagged individually with wing bands, and housed in floor pens with fresh wood shavings in an environmentally controlled room under standard management. Chickens had free access to tap water and an unmedicated mash corn-soybean starter diet (crude protein 21.5%) that meets or exceeds the nutrient requirements of the NRC recommendations throughout the study.

On day 10, a total of 100 chickens were weighed individually after overnight fasting and transferred to 17 battery cages with 5–6 chickens per cage for experimental infection. The bottom of each battery cage was covered with a cardboard box throughout the entire experiment to encourage the recycling of parasites. Upon transfer, 90 chickens in 15 cages were inoculated orally with 5,000 sporulated oocysts of *E. maxima* strain M6 (kindly provided by Dr. John R. Barta, University of Guelph, Canada; Al-Badri and Barta, 2012) in 1mL saline, while the remaining 10 chickens in two cages were gavaged with 1mL saline and served as mock-infected controls. The *netB-* and *tpeL-*positive *C. perfringens* strain Brenda B (kindly provided by Dr. Lisa Bielke at the Ohio State University, Columbus, OH, United States; Latorre et al., 2018) was sequentially passaged in chopped cooked meat medium and fluid thioglycollate medium. On day 14, approximately 4×10^8^CFU of *C. perfringens* in 2mL of overnight culture was administered by oral gavage, while mock-infected control chickens were orally gavaged only with 2mL fluid thioglycollate medium. To minimize cross-contamination, mock-infected chickens were put on the top level of the battery cages and always handled the first.

Animals were observed twice daily till day 17. Chickens reluctant to move were euthanized to minimize undue suffering. Survival rate (%) and weight loss (%) of infected chickens during days 10–17 were calculated relative to healthy controls. On day 17, all surviving chickens were weighed individually and then sacrificed *via* CO_2_ asphyxiation. Lesions in the small intestine were examined and scored on a scale of 0–6 as described (Shojadoost et al., 2012), where score 0 indicated a healthy intestine, score 1 was manifested by thin intestinal walls or removable fibrin on the mucosal surface, and scores 2, 3, and 4 referred to the lesions of 1–5, 6–15, and >16 foci, respectively, while score 5 was denoted by the presence of extended necrosis of 2–3cm in length and score 6 displayed extensive and diffuse necrosis typical of field cases. Additionally, approximately 0.5g of the digesta in the proximal ileum were collected and stored at −80°C for microbial genomic DNA extraction.

### Bacterial DNA Isolation and Estimation of Total Bacteria

Bacterial genomic DNA was extracted from the ileal digesta using the ZR Fecal DNA MicroPrep Kit (Zymo Research, Irvine, CA, United States), quantified with Nanodrop, and then diluted to 10ng/μL with nuclease-free water. The 16S rRNA gene copy number in the ileal microbiome was measured using Femto Bacterial DNA Quantification Kit (Zymo Research, Irvine, CA, United States). Total bacterial 16S rRNA gene copy number/g digesta were calculated based on the standard curve generated using the primers specific for the V4 region of the 16S rRNA gene and genomic DNA standards of *Escherichia coli* strain JM109 provided in the kit. Because five most dominant bacteria represented a great majority (89–99%) of the total bacterial population in the ileum, total bacterial genome copy number/g digesta were further estimated as: total 16S rRNA gene copy number/g digesta×(A1/B1+A2/B2+A3/B3+A4/B4+A5/B5)/C, where A1–A5 are relative abundances (%) of five individual bacteria, B1–B5 are 10, 4, 6, 6, and 7, which represent the 16S rRNA gene copy number/genome of *C. perfringens* (Shimizu et al., 2002), group A *Lactobacillus* (Altermann et al., 2005), group B *Lactobacillus* (Pridmore et al., 2004), *Lactobacillus reuteri* (Heavens et al., 2011), and *Escherichia*/*Shigella* (Vetrovsky and Baldrian, 2013), respectively.

### 16S rRNA Gene Sequencing and Bioinformatics Analysis

Microbial DNA of the ileal digesta was also subjected to 16S rRNA amplicon sequencing as described (Wang et al., 2019b). Briefly, the V4 region of the bacterial 16S rRNA gene was amplified by PCR using the primers 515F (GTGCCAGCMGCCGCGGTAA) and 806R (GGACTACHVGGGTWTCTAAT) and sequenced on an Illumina MiSeq platform using a dual-index 250-bp paired-end sequencing approach as described (Kozich et al., 2013). The negative control and Mock Community [ZymoBIOMICS™ Microbial Community Standard (Zymo Research, Irvine, CA, United States)] were also included in the library construction and each MiSeq sequencing run for quality control. Raw sequencing reads were then processed with QIIME2 (v.2019.10; Bolyen et al., 2019). Sequencing reads were sequentially demultiplexed, filtered, and denoised with Deblur, generating amplicon sequence variants (ASVs), which differ at the single nucleotide level (Amir et al., 2017). The taxonomies of individual ASVs were initially classified using Naive Bayes classifier trained on the Greengenes 13_8 99% OTU reference database and further confirmed and reclassified, if necessary, based on a more recent EzBioCloud 16S database (Version 20200513; Yoon et al., 2017). ASVs present <5% of samples were removed from the downstream analysis. Moreover, the microbiota data were normalized by cumulative-sum scaling using the R “metagenomeSeq” package to correct biases in sampling depth (Paulson et al., 2013).

Alpha and beta diversity of bacterial community were computed using the “phyloseq” package (v.1.30.0) in R (Mcmurdie and Holmes, 2013). Observed ASVs, Pielou’s evenness, and Shannon diversity index were used to indicate the richness, evenness, and overall alpha diversity, while beta diversity was visualized using principal coordinates analysis (PCoA) plots based on unweighted and weighted UniFrac distances. The composition of the ileal microbiota was indicated by relative abundance of bacterial taxa at phylum, order, family, genus, and ASV levels. For those relatively abundant bacterial taxa that were commonly present in a minimum of 20% of the samples, linear discriminant analysis (LDA) effect size (LEfSe) analysis (Segata et al., 2011) was performed to identify their differential enrichment with *p*<0.05, logarithmic LDA score of >2.0, and the all-against-all strategy.

Furthermore, the bacterial taxa that were present in >20% of samples were further calculated for their association with the lesion score and weight loss using Spearman correlation and the corr.test function in R “psych” package (v.1.9.12.31; https://personality-project.org/r/psych/). Fold changes in relative abundance of taxa significantly associated with NE severity were further calculated relative to mock-infected healthy controls, followed by log2 transformation and displayed in Heatmap using the R package “pheatmap” (v.1.0.12). Additionally, differential enrichment of Kyoto Encyclopedia of Genes and Genomes (KEGG) metabolic functions of the ileal microbiota between mild and severe NE chickens was predicted using Phylogenetic Investigation of Communities by Reconstruction of Unobserved States (PICRUSt2; Douglas et al., 2020).

### Statistical Analysis and Data Visualization

Statistical analysis was conducted in GraphPad Prism (GraphPad Software, La Jolla, CA, United States) or RStudio (v.1.2.1578; RStudio, Boston, MA, United States). Significance was determined using parametric or non-parametric method depending upon data normality following Shapiro-Wilk test. One-way ANOVA and Tukey *post-hoc* test were applied to compare animal growth performance, lesion score, and the number of total bacteria among different groups of animals, while Kruskal-Wallis and pairwise Wilcoxon rank-sum tests were used to compare alpha diversity and relative abundance of the ileal microbiota. The survival rate of chickens was analyzed using the log-rank test. Significance of weighted and unweighted Unifrac distances was evaluated by permutational multivariate ANOVA (PERMANOVA) using the Adonis function of the “vegan” package (v.2.5.6) under the default setting (permutations=999). Spearman correlation was corrected with the Benjamini-Hochberg procedure to control the false discovery rate (FDR). Two-sided White’s non-parametric *t*-test and FDR correction were performed with PICRUSt2-predicted KEGG pathways in the Statistical Analysis of Metagenomic Profiles (STAMP) software package (v2.1.3; Parks et al., 2014). *p*<0.05 or FDR<0.05 was considered statistically significant. Graphs in R were made with the “ggplot2” package (v.3.3.0; Wickham, 2016).

## Results

### Characterization of a Chicken Model of Clinical NE

**Figure 1:**
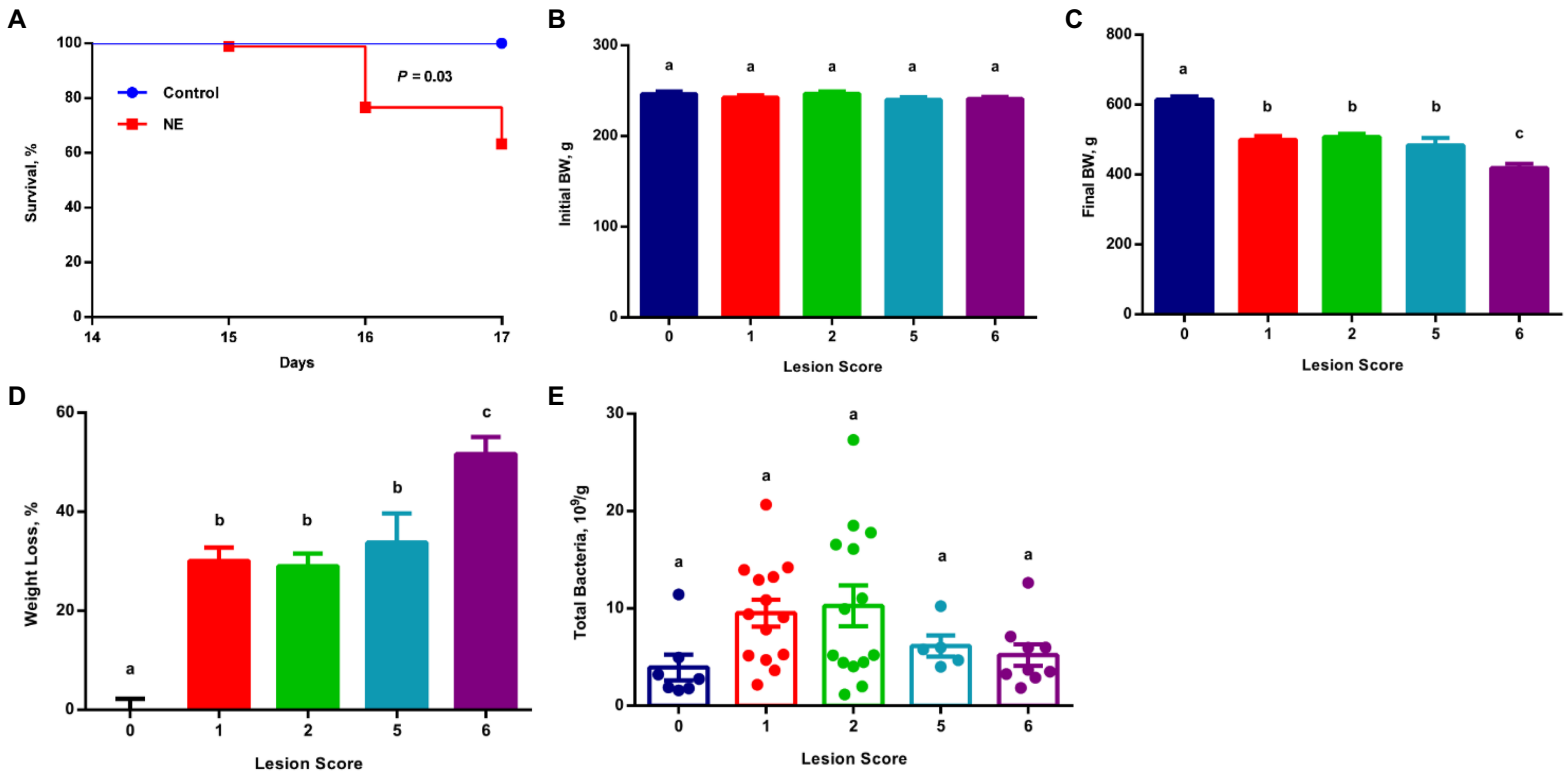
Survival rate, growth performance, and total bacterial population among chickens with varying severities of necrotic enteritis (NE). A group of 90 male Cobb broilers were infected sequentially with *Eimeria maxima* on day 10 and *Clostridium perfringens* on day 14, while another 10 chickens were mock-infected as negative controls. Animals were separated into five groups according to their respective intestinal lesion scores measured on day 17. Animal survival was recorded daily between days 14 and 17 **(A)** and was compared between infected and mock-infected healthy animals using the log-rank test. Body weights of individual animals were recorded on day 10 **(B)** and day 17 **(C)**. Weight loss (%) of infected chickens during days 10–17 **(D)** was calculated relative to healthy controls. Total bacteria number in the ileal digesta **(E)** was also estimated using qPCR. All data were expressed as means±SEM. Different superscripts denote significance (*p*<0.05) based on one-way ANOVA and Tukey’s *post-hoc* test.

Cobb broiler chickens quickly developed typical clinical signs of NE, such as lethargy, anorexia, and diarrhea after challenge with *E. maxima* M6 (Al-Badri and Barta, 2012; Wang et al., 2019a; Bansal et al., 2020), followed by a second challenge with *netB*- and *tpeL*-positive *C. perfringens* strain Brenda B. Among 90 infected chickens, 33 were euthanized due to NE illness or death by day 17 showing a mortality rate of 36.7% (Figure 1A). All surviving animals were sacrificed on day 17, and the severity of lesions in the small intestine was evaluated on a scale of 0–6 as described (Shojadoost et al., 2012). While all 10 mocked infected animals had healthy intestines and received a score of 0, all surviving infected chickens displayed a varying degree of lesions mostly in the jejunum and proximal ileum. Fifteen infected chickens were scored 1, and 17 were scored 2, with both groups showing no obvious or fewer than five foci of lesions, while five chickens received a score of 5 and 13 received a score of 6 showing larger than 2–3cm of necrotic patches. Another two chickens were scored 3 and two chickens scored 4 with more than five loci of lesions, but they were excluded from subsequent analyses due to the small sample size.

All chickens were separated into different groups according to their respective lesion scores. Although all groups of chickens started with similar body weight on day 10 (Figure 1B), obvious growth retardation was observed proportional to the severity of the lesions (Figure 1C). Relative to mock-infected controls (score 0), approximately 30% weight loss occurred to the chickens with mild lesions (score 1), while >50% weight loss happened to the chickens with severe lesions (score 6; Figure 1D). Collectively, weight loss and the mortality rate were consistent with a typical clinical episode of NE in the field.

To examine the total bacterial population in the ileum of mild and severe NE, the proximal ileal contents were collected from individual animals and total bacterial counts were estimated using standard curve-based qPCR. The ileal bacterial population increased in response to NE. In comparison with mock-infected healthy (score 0) chickens harboring approximately 3.9×10^9^ total bacteria/g digesta, the total number of bacteria increased by approximately 2.5-fold in NE chickens with a lesion score of 1 or 2, and 1.5-fold in chickens with a lesion score of 5 or 6 (Figure 1E).

### Shifts in the Ileal Microbiota Composition in Response to NE

**Figure 2:**
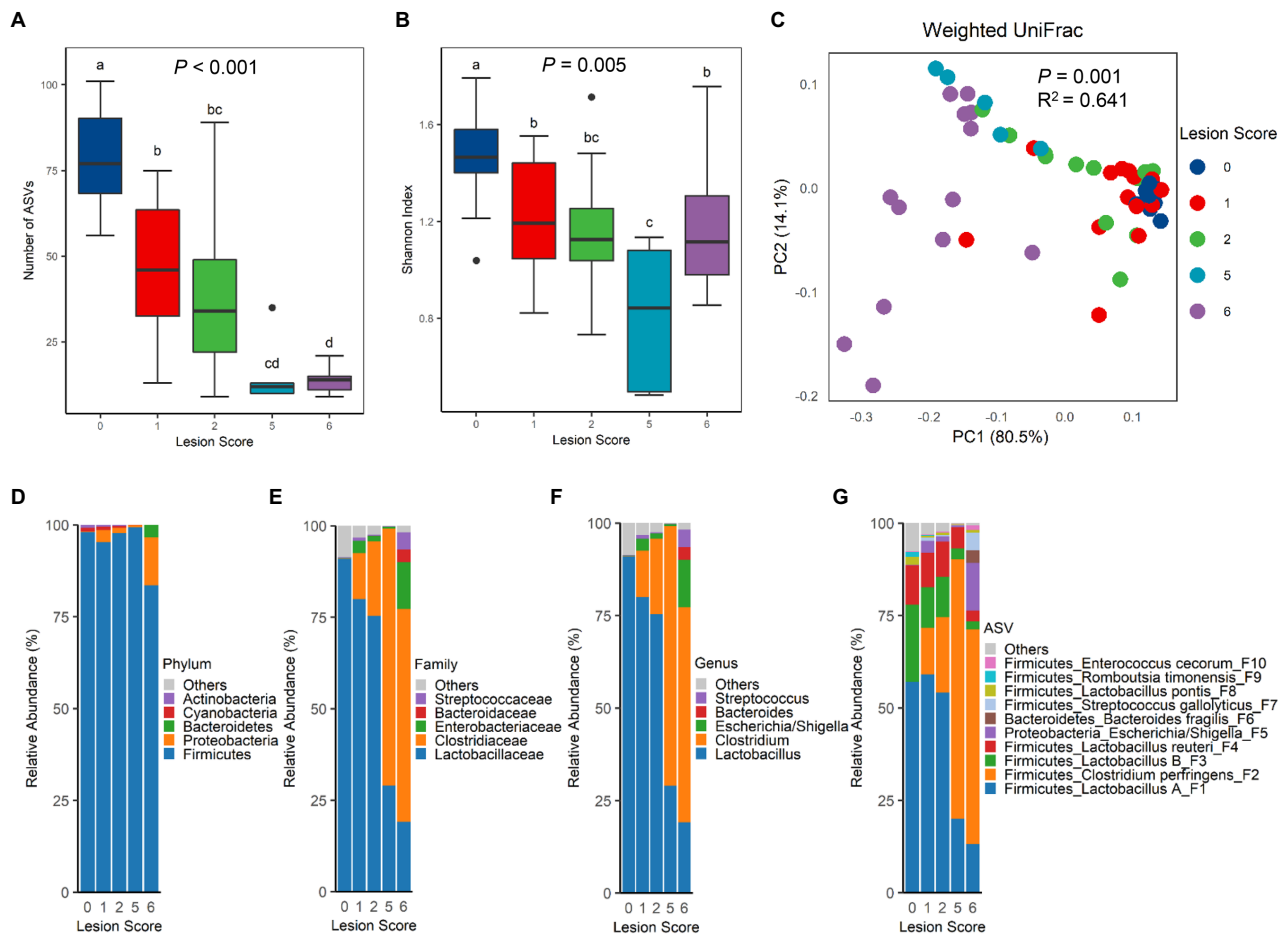
Diversity and composition of the ileal microbiota among chickens with varying severities of NE. A group of 90 male Cobb broilers were subjected to NE, while another 10 chickens in the control group were mock-infected. Animals were divided into five groups based on lesion scores. The number of observed amplicon sequence variants (ASVs; **A**) and Shannon index **(B)** of the ileal microbiome were estimated and visualized using box and whisker plots. Each box indicates median, 25 and 75th percentiles, while whiskers extend to 1.5 interquartile range. Significance was measured using the Kruskal-Wallis test, and pairwise comparisons were implemented using the Wilcoxon rank sum test. Different superscripts denote significance (*p*<0.05) in pairwise comparisons. **(C)** Principal coordinates analysis (PCoA) plots of weighted UniFrac distances. Significance was determined using permutational multivariate ANOVA (PERMANOVA). Relative abundances of top five phyla **(D)**, top five families **(E)**, top five genera **(F)**, and top 10 ASVs **(G)** in the ileal microbiota were shown.

To evaluate the compositional shift in the intestinal microbiota of chickens in response to NE, 16S rRNA gene sequencing was performed with the bacterial DNA isolated from the ileal content of NE-afflicted and mock-infected control chickens. A total of 1,590,185 raw sequencing reads were obtained from 66 samples and subjected to the QIIME 2 pipeline (Bolyen et al., 2019). After Deblur (Amir et al., 2017), 1,120,661 clean reads were retained with 16,979±4,839 (SD) sequences per sample. A total of 164 unique ASVs were obtained after removal of those present in <5% of samples. The richness of the ileal microbiota, as measured by the number of observed ASVs, was progressively decreased in chickens as NE was exacerbated (Figure 2A). The median ASV number was reduced from 77 in mock-infected control (score-0) chickens to 12–14 ASVs in score-5 or -6 chickens. Shannon index, which measures the overall alpha diversity of the bacterial community, was also gradually decreased (Figure 2B). However, Pielou’s evenness index remained largely unchanged among healthy chickens and chickens with lesion scores of 1, 2, and 5, although score-6 chickens showed a significantly higher evenness than other groups (*p*=0.002; Supplementary Figure 1A).

The differences in the ileal microbiota community among chickens of varying NE severities were further evaluated by unweighted and weighted UniFrac metrics. There was an obvious segregation of the ileal microbiota among different groups of chickens in both weighted UniFrac (*p*=0.001, *R*^2^=0.641; Figure 2C) and unweighted UniFrac (*p*=0.001, *R*^2^=0.350; Supplementary Figure 1B). Pairwise PERMANOVA test (Anderson, 2001) further revealed a significant difference (*p*<0.05) in both unweighted and weighted UniFrac in nearly all pairs, except for the ones between score-1 and -2 chickens and between score-2 and -5 chickens in unweighted UniFrac (Supplementary Table 1), suggesting that there were clear differences in the microbiota composition among nearly all groups of chickens. It is noted that the microbiota variation was progressively increased among individual animals following infection, while mock-infected chickens in the control group had a relatively uniform microbiota composition (Figure 2C; Supplementary Figure 1B).

Top 20 bacterial ASVs accounted for 95.2–99.9% of the bacterial population in the ileum of all chickens (Supplementary Table 2). Lactobacilli amounted to >90% of bacteria in the ileum of healthy, mock-infected chickens in our study (Supplementary Table 3). A drastic change in the microbiota composition occurred in response to NE. At the phylum level, a significant decrease in Firmicutes (FDR=0.007) and an increase in Proteobacteria (FDR=0.005) were observed in score-6 chickens, relative to other groups of chickens (Figure 2D; Supplementary Table 3). Although there was no change in the Bacteroidetes population, Cyanobacteria and Actinobacteria were gradually decreased along with the exacerbation of NE (Supplementary Table 3). At the family (Figure 2E) and genus (Figure 2F) levels, NE severity-dependent decline in the *Lactobacillaceae* family and the *Lactobacillus* genus in particular was evident, while the *Clostridiaceae* family and particularly the *Clostridium* genus showed a gradual increase. Among the top 10 families and top 10 genera, most were significantly altered by NE, except for *Bacteroides* and *Streptococcus* (Supplementary Table 3). At the ASV level, *Lactobacillus* was mostly comprised of four ASVs, group A *Lactobacillus* (F1), group B *Lactobacillus* (F3), *L. reuteri* (F4), and *L. pontis* (F8), while *Clostridium* was mainly represented by *C. perfringens* (F2; Figure 2G). While all major lactobacilli in the ileum were progressively suppressed by NE, *C. perfringens* experienced a drastic increase. *Lactobacillus* collectively dominated the ileal microbiota accounting for 91.0% in healthy chickens, but was gradually decreased to 80.0, 75.3, 29.1, and 19.1% in infected chickens with a lesion score of 1, 2, 5, and 6, respectively (Supplementary Table 3). On the contrary, *C. perfringens* accounted only for 0.02% of the total bacterial population in healthy chickens, but quickly expanded to 12.5, 20.5, 70.2, and 58.1% in score-1, -2, -5, and -6 chickens, respectively, becoming the most predominant bacteria in the ileum of severely infected chickens (Figure 2G; Supplementary Table 3). In fact, 15 out of the top 20 bacterial ASVs were significantly altered by NE (FDR<0.05; Supplementary Table 3).

### Differential Enrichment of Bacteria in Chickens With Mild and Severe NE

**Figure 3:**
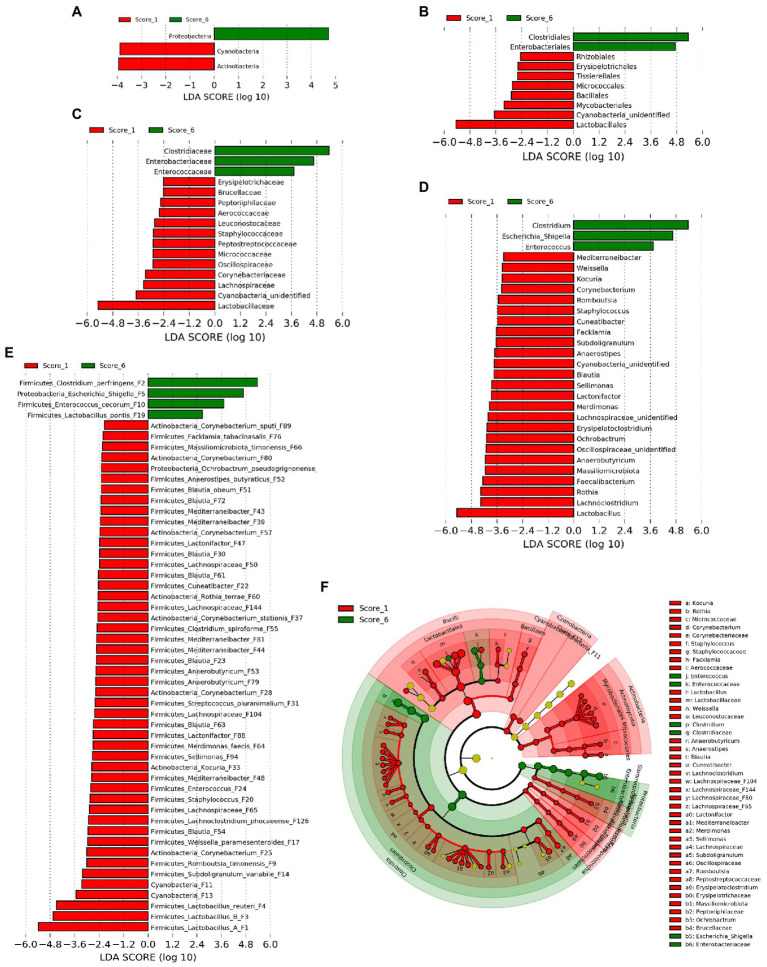
Differential enrichment of the ileal bacteria between chickens with mild and severe NE. After induction of NE, the ileal digesta of broilers were collected and subjected to 16S rRNA gene sequencing. Intestinal lesions of individual animals were scored. Linear discriminant analysis (LDA) effect size (LEfSe) analysis was performed to identify differentially abundant bacteria at the phylum **(A)**, order **(B)**, family **(C)**, genus **(D)**, and ASV levels **(E)** between score-1 mild and score-6 severe NE chickens with cut-offs *p*<0.05 and LDA score>2.0. **(F)** Phylogenetic relationships of differentially enriched bacteria. Differentially enriched bacterial taxa were represented by rings with the phylum shown in the outermost ring and the species in the innermost ring.

In order to identify ileal bacterial taxa that were differentially enriched between chickens with mild (score 1) and severe NE (score 6), LEfSe analysis (Segata et al., 2011) was performed among bacterial taxa that were commonly present in >20% of samples to exclude those rare bacteria. At the phylum level, Proteobacteria was enriched in score-6 chickens, while Actinobacteria and Cyanobacteria were more abundant in score-1 chickens (Figure 3A). At the order level, Clostridiales and Enterobacteriales were enriched in severe NE, while eight other orders such as Lactobacillales were more abundant in mild NE (Figure 3B). Among a total of 18 families and 31 genera detected, 16 families (Figure 3C) and 28 genera (Figure 3D) were enriched in either score-1 or score-6 chickens. While three genera, including *Clostridium*, *Escherichia*/*Shigella*, and *Enterococcus* were increased in severe NE, *Lactobacillus*, *Lachnoclostridium*, and *Rothia* were among those that were greatly diminished in response to severe NE (Figure 3D). Among a total of 64 bacterial ASVs that were shared among >20% samples, 52 ASVs were differentially enriched (Figure 3E). While 48 ASVs were found to be more abundant in mildly infected chickens, four ASVs were enriched in severely infected chickens. *C. perfringens* (F2), an *Escherichia*/*Shigella* member (F5), and *Enterococcus cecorum* (F10) were strongly enriched in severe NE, whereas three *Lactobacillus* species, including group A *Lactobacillus* (F1), group B *Lactobacillus* (F3), and *L. reuteri* (F4) were among the most enriched bacteria in mild NE (Figure 3E).

Phylogenetically, all differentially abundant bacteria belonged to four phyla, including Firmicutes, Proteobacteria, Actinobacteria, and Cyanobacteria. Three major bacteria that were enriched in chickens with severe NE belonged to three distinct bacterial orders, namely Clostridiales (*C. perfingens* F2), Enterobacteriales (*Escherichia*/*Shigella* F5), and Lactobacillales (*E. cecorum* F10; Figure 3F). On the other hand, major bacteria that were diminished in severely infected chickens included four members of Lactobacillales (*Lactobacillus* F1, F3, and F4, and *Weissella paramesenteroides* F17), one member of Bacillales (*Staphylococcus* F20), and multiple members of Clostridiales in the *Lachnospiraceae*, *Oscillospiraceae*, and *Peptostreptococcaceae* families (Figure 3F). Notably, multiple members of Actinobacteria including several species of *Corynebacterium* [(F25, F28, F37, F57, F80, and F89), *Kocuria* (F33), and *Rothia* terrae (F60)] as well as two highly related Cyanobacteria (F11 and F13) were also reduced in severely infected chickens (Figure 3F).

To reveal the microbiota changes in response to a mild NE infection and also to identify those bacteria that were hypersensitive to and quickly abolished by NE, LEfSe analysis was performed again to compare the ileal microbiota compositions between mock-infected healthy (score-0) and mildly infected (score-1) chickens. Similar to the pattern observed between mild and severe NE, *C. perfringens* (F2) and *Escherichia* (F68) were found to be enriched in mildly infected chickens, while *Lactobacillus* (F3, F8, and F19), *Staphylococcus* (F20), and multiple species in the families of *Lachnospiraceae* (e.g., F22, F23, F30, and F32), *Oscillospiraceae* (e.g., F45 and F95), and *Peptostreptococcaceae* (e.g., F9) were diminished in response to mild NE infection (Supplementary Figure 2).

### Correlation Between the Ileal Microbiota Profile and the Severity of NE

**Figure 4:**
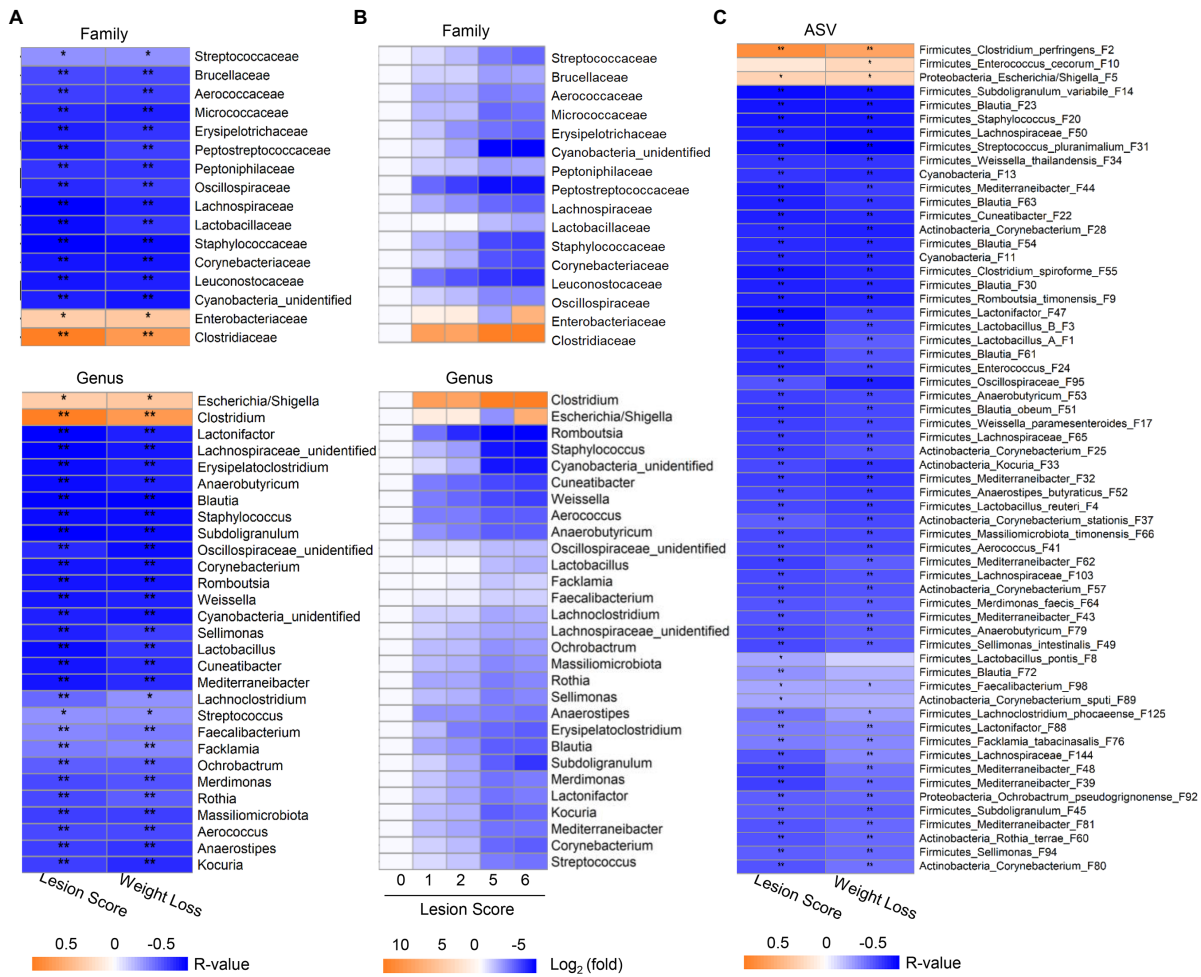
Correlation between relative abundance of the ileal bacteria and the severity of NE. The Spearman correlation analysis was performed between the ileal microbiota profile and the intestinal lesion score and weight loss during days 10–17. **(A)** Spearman correlation at the family and genus levels. **(B)** Heatmap showing log2 transformations of the fold changes of the ileal bacteria among different groups of NE at the family and genus levels relative to score-0 healthy controls. **(C)** Spearman correlation at the ASV level. Significance of Spearman correlation analysis was corrected with the Benjamini-Hochberg procedure. ^*^FDR<0.05, ^**^FDR<0.01.

Spearman rank correlation was further employed to analyze the correlation between the relative abundance of the ileal microbiota and the lesion score and weight loss, respectively. Among 18 bacterial families and 31 genera that were commonly present in >20% of samples, 16 families and 29 genera showed a significant correlation (FDR<0.05) with the two phenotypic parameters (Figure 4A). While *Clostridiaceae*/*Clostridium* and *Enterobacteriaceae*/*Escherichia*-*Shigella* were positively correlated with NE severity, all other bacterial families and genera showed a strong negative correlation. The prevalence of most bacteria, such as *Streptococcaceae*/*Streptococcus*, *Staphylococcaceae*/*Staphylococcus*, *Lactobacillaceae*/*Lactobacillus*, and *Oscillospiraceae*/*Subdoligranulum* progressively declined along with the severity of NE (Figure 4B). In addition, two genera of Actinobacteria including *Kocuria* and *Corynebacterium* as well as an unclassified Cyanobacteria genus showed a clear gradual decrease as NE became more severe. Moreover, different bacteria apparently varied in their sensitivity to NE. *Leuconostocaceae*/*Weissella*, *Lachnospiraceae*/*Cuneatibacter*, and *Peptostreptococcaceae*/*Romboutsia* were drastically reduced in response to even mild NE and remained diminished in severe NE (Figure 4B).

**Figure 5:**
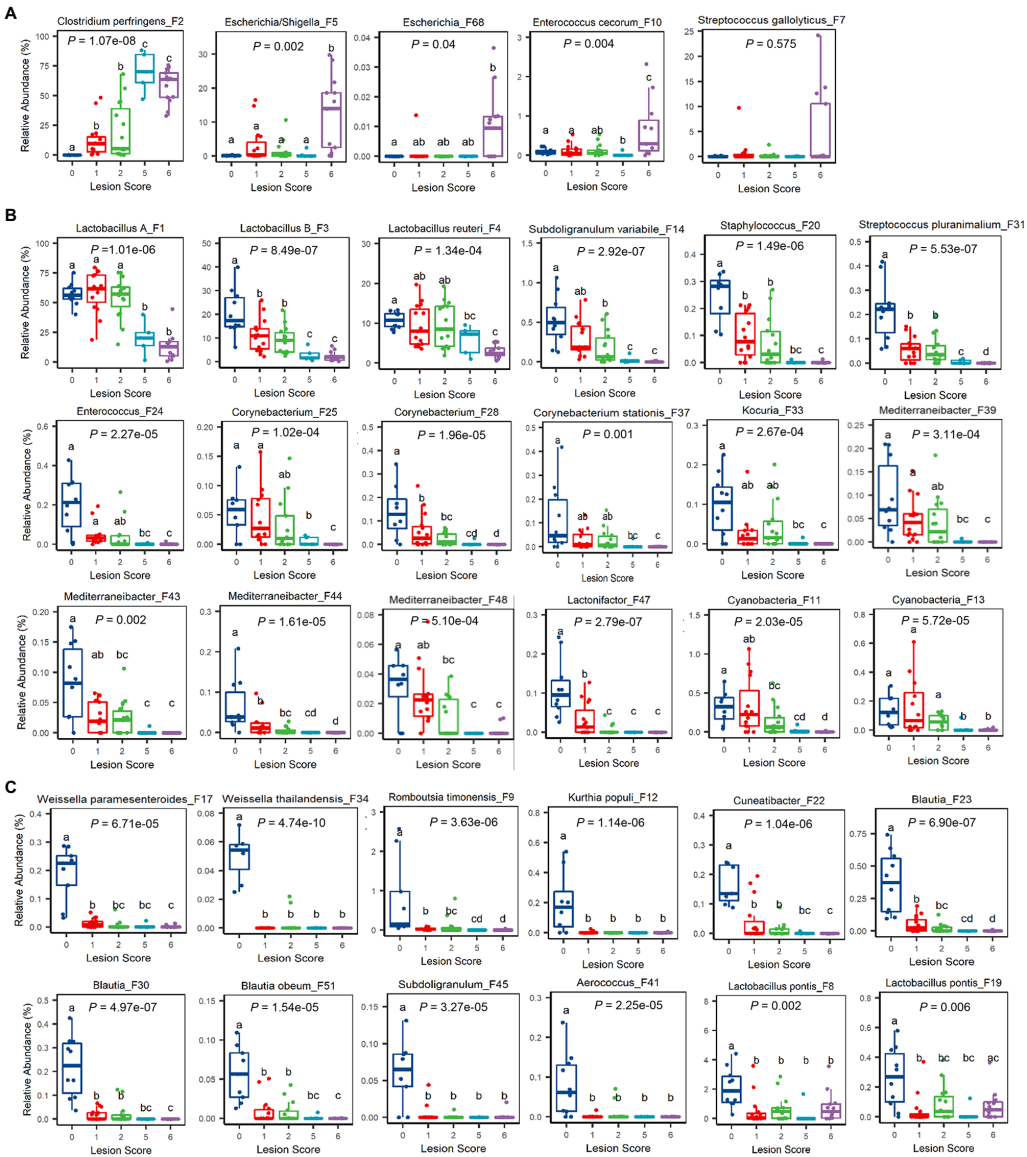
Relative abundances of representative ileal bacteria showing a significant correlation with the severity of NE. Ileal bacteria were significantly enriched **(A)**, progressively declined **(B)**, or abruptly abolished **(C)** in response to NE. In the box and whisker plots, each box indicates median, 25 and 75th percentiles, while whiskers extend to 1.5 interquartile range. Significance was measured using the Kruskal-Wallis test and indicated on the top of each plot. Pairwise comparisons were further implemented using the Wilcoxon rank sum test, and the significance (*p*<0.05) was denoted by different superscripts.

At the ASV level, 60 out of 64 bacterial ASVs that were shared among >20% samples showed a significant correlation with NE severity indicated by the lesion score or weight loss (FDR<0.05; Figure 4C). All but three bacterial ASVs (*C. perfringens* F2, *Escherichia*/*Shigella* F5, and *E. cecorum* F10) were negatively correlated with the severity of NE. Quantitatively, among the four most dominant bacterial species in the ileum, *C. perfringens* (F2) increased from 0.02% in mock-infected healthy chickens (score-0) to approximately 60–70% in severely infected chickens (score-5 and -6; Figure 5A), while group A and group B *Lactobacillus* (F1 and F3) as well as *L. reuteri* (F4) were gradually decreased in NE chickens (Figure 5B). Collectively, three dominant *Lactobacillus* species were reduced from approximately 91% in healthy, mock-infected chickens to 19% in score-6 chickens. In addition, a number of top 50 bacteria, such as *Subdoligranulum variabile* (F14), *Staphylococcus* (F20), and *Streptococcus pluranimalium* (F31), *Enterococcus* (F24), several members of *Corynebacterium* (F25, F28, and F37) and *Mediterraneibacter* (F39, F43, F44, and F48), and *Lactonifactor* (F47) were progressively diminished as NE was exacerbated (Figure 5B). Two related cyanobacteria (F11 and F13) also gradually declined along with NE severity (Figure 5B).

It is noteworthy that most of the differentially enriched bacteria between mild and severe NE were strongly correlated with NE severity. Among those bacteria showing a strong correlation with NE, a closer examination revealed multiple bacteria, such as two *Weissella* species (F17 and F34), *Cuneatibacter* (F22), several *Blautia* members (F23, F30, and F51), *Aerococcus* (F41), and *Subdoligranulum* (F45) were hyper-sensitive to NE infection, diminished quickly in mildly infected chickens, and vanished essentially in chickens with severe NE (Figure 5C). Interestingly, in contrast with predominant group A and B *Lactobacillus* and *L. reuteri*, which showed a gradual decline in infected chickens, less dominant *L. pontis* (F8 and F19) appeared to be very sensitive to NE, with a substantial reduction even during a mild infection (Figure 5C). On the other hand, several bacteria, including *Escherichia*/*Shigella* (F5), *Escherichia* (F68), and *E. cerorum* (F10) were significantly enriched only in severely infected, score-6 chickens (Figure 5A). *Streptococcus gallolyticus* (F7) remained largely unaffected by NE, even with a numerical increase in abundance in severely infected chickens (Figure 5A).

It is important to note that not all related bacteria responded to NE uniformly. Obvious variations within several genera existed. Besides an obvious differential response to NE among four *Lactobacillus* species (group A and group B *Lactobacillus*, *L. reuteri*, and *L. pontis*), *E. cecorum* (F10) was increased during extremely severe NE, while a less abundant *Enterococcus* species (F24) was gradually abolished by NE. *Streptococcus gallolyticus* (F7) was largely unaffected by NE, but another *Streptococcus* species (*S. pluranimalium* F31) showed a progressive decline. *Subdoligranulum variabile* (F14) was gradually diminished by NE, while another *Subdoligranulum* species (F45) became rapidly disappeared.

**Figure 6:**
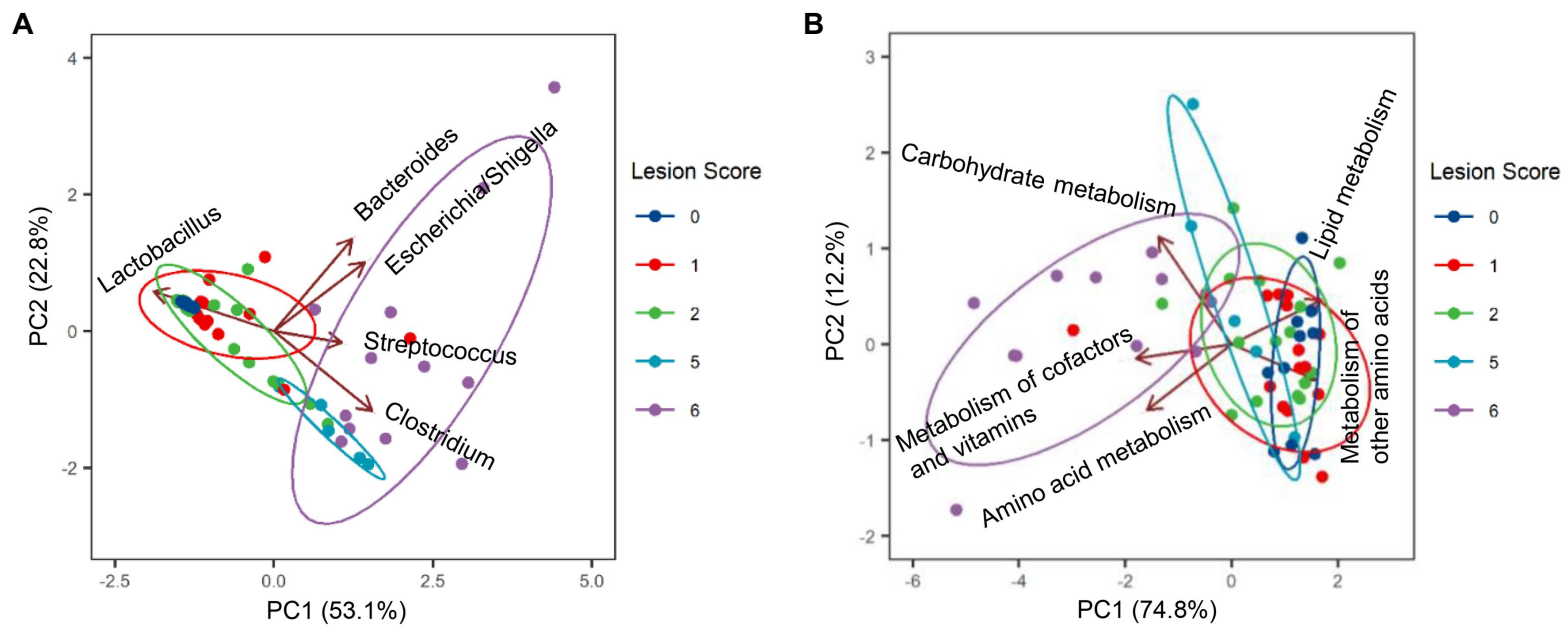
Biplots of principal component analysis showing relative contributions of top five genera **(A)** and top five predicted Kyoto Encyclopedia of Genes and Genomes (KEGG) pathways **(B)** to the divergence of the ileal microbiota in response to NE. Each biplot line shows the direction of the change, with the length of each line indicating the degree of correlation with ordination axes.

Among the most dominant genera, *Clostridium* (represented by *C. perfringens* F2) was the primary driver for the microbiota profile of severely infected chickens, whereas *Lactobacillus* (represented by F1, F3, and F4) was primarily responsible for maintaining the microbiota of healthy chickens (Figure 6A). While *Bacteroides* (*B. fragilis* F6) played no role in explaining the microbiota differences, *Streptococcus* (*S. gallolyticus* F7) and *Escherichia*/*Shigella* (F5) had a minimum impact (Figure 6A).

### Microbial Functional Changes Between Mild and Severe NE

**Figure 7:**
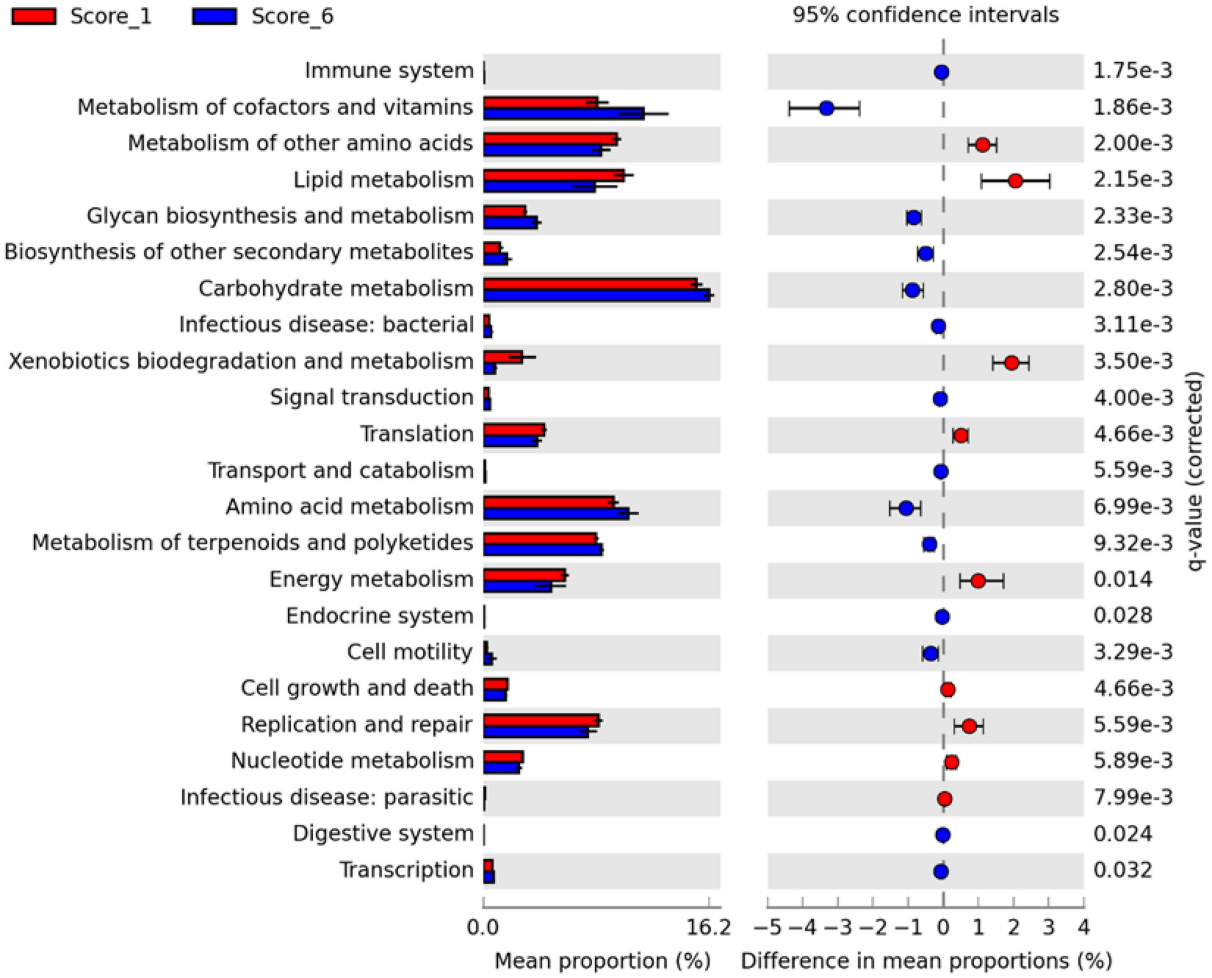
Differential enrichment of predicted KEGG pathways between mild and severe NE. Functional potentials of ileal microbiota of score-1 and score-6 chickens were predicted using PICRUSt2 and level-2 KEGG pathways were compared in Statistical Analysis of Metagenomic Profiles (STAMP). Each bar represents a mean proportion (%) and SEM of a predicted KEGG pathway. The 95% CIs of the differences in mean proportions between score-1 and score-6 chickens were also shown. Significance was determined by two-sided White’s non-parametric *t*-test, corrected with the Benjamini-Hochberg procedure, and indicated as *q* values on the right. Only those pathways showing *q*<0.05 were displayed.

To evaluate possible alterations in the microbiota function between mild and severe NE, PICRUSt2 (Douglas et al., 2020) was used to predict the functional potential of the ileal microbiota in score-1 and score-6 chickens based on 16S rRNA gene sequencing reads. Among 28 level-2 KEGG pathways identified, 23 were found to be significantly different between score-1 and score-6 chickens (FDR<0.05; Figure 7). Relative to mildly infected chickens, metabolisms of cofactors, vitamins, amino acids, and carbohydrates were predicted to be heightened in severely infected chickens, while metabolisms of lipids, energy, xenobiotics, and certain amino acids were predicted to be significantly suppressed (Figure 7). Among the top five pathways, metabolism of cofactors and vitamins, amino acid metabolism, and carbohydrate mechanism were predicted to be major drivers for the microbiota of severely infected chickens, while lipid metabolism and metabolism of other amino acids were predicted to be responsible for maintaining the homeostasis of the ileal microbiota of healthy chickens in the context of NE infection (Figure 6B).

Between score-1 and score-6 chickens, 107 out of 149 level-3 KEGG pathways were statistically different (FDR<0.05; Supplementary Figure 3). Relative to score-1 chickens, biosynthesis of aromatic amino acids (phenylalanine, tyrosine, and tryptophan) and branched-chain amino acids (leucine, isoleucine, and valine) and metabolism of the majority of amino acids were predicted to be obviously increased, while lysine biosynthesis and D-glutamine/D-glutamate metabolism were predicted to be suppressed in severely infected chickens (Supplementary Figure 3). Metabolisms of cofactors and vitamins, such as biotin, lipoic acid, and nicotinate/nicotinamide, were predicted to be enhanced in severe NE. Interestingly, the ileal microbiota of severely infected chickens was predicted to be less active in degrading xenobiotic compounds, such as toluene, naphthalene, benzoate, caprolactam, and dioxin than that of mildly infected chickens (FDR<0.05; Supplementary Figure 3). Primary and particularly secondary bile acid biosynthesis was also predicted to be significantly enriched in chickens with mild NE. Moreover, biosynthesis of antibiotics, such as vancomycin group antibiotics, streptomycin, penicillin, cephalosporin, and siderophore group nonribosomal peptides was predicted to be significantly enhanced in score-6 NE chickens (FDR<0.01; Supplementary Figure 3).

## Discussion

### A Drastic Shift of the Bacterial Dominance in the Ileum of NE Chickens

Necrotic enteritis is known to cause intestinal dysbiosis (Antonissen et al., 2016; Sun and Jia, 2018). However, the magnitude and the type of dysbiosis varied greatly among different studies. Subtle to moderate shifts in the composition of the jejunal, ileal, or cecal microbiota have been reported (Stanley et al., 2012; Lin et al., 2017; Latorre et al., 2018; Xu et al., 2018; Zhang et al., 2018; Bortoluzzi et al., 2019; Hernandez-Patlan et al., 2019; Kiu et al., 2019; Yang et al., 2019; Lu et al., 2020). Such inconsistencies are largely due to the heterogeneity in disease severity among individual NE-afflicted animals examined in a study and variations in the severity of NE induced in chickens among different studies. No research has examined the microbiota shift in association with the severity of NE. In this study, we have experimentally infected a large group of broiler chickens with NE and separated them based on disease severity. After assessing relative and absolute changes in the ileal microbiome following infection, we have revealed for the first time how relative abundances of most bacteria were altered in response to NE and how dysbiosis became more pronounced as NE was exacerbated.

The most dramatic alteration was a reciprocal shift in dominance between lactobacilli and *C. perfringens* in the ileum. While predominant lactobacilli progressively declined to 19% in severely infected chickens, *C. perfringens* quickly expanded to 58–70% in severe infections. It is worth noting that several distinct species of *Lactobacillus* were detected in this study. In healthy ileum, the most abundant group A *Lactobacillus* were represented by three highly related species, *L. acidophilus*, *L. crispatus*, and *L. gallinarum* (Fujisawa et al., 1992; Guan et al., 2003), while the second most abundant group B *Lactobacillus*, which were represented by *L. johnsonii* and *L. gasseri* (Fujisawa et al., 1992; Guan et al., 2003). Two less abundant *Lactabocillus* species including *L. reuteri* and *L. pontis* were also present in healthy ileum. Although we could not separate bacteria within group A or B *Lactobacillus* due to sequencing of only the V4 region of the 16S rRNA gene, it is entirely possible that multiple species of each group could co-exist in the ileum. In fact, *L. acidophilus*, *L. crispatus*, *L. gallinarum*, *L. johnsonii*, *L. gasseri*, *L. reuteri*, and *L. pontis* have all been detected in the small intestine of chickens (Guan et al., 2003; Bjerrum et al., 2006). *Lactobacillus salivarius* and *L. aviarius* were among major *Lactobacillus* species that were commonly isolated in chickens (Guan et al., 2003; Bjerrum et al., 2006; Wang et al., 2014), but they were not detected in this study, presumably due to environmental variations among different studies.

Importantly, we revealed that different *Lactobacillus* species showed different sensitivities to the colonization of *C. perfringens*, underscoring the importance of differentiating *Lactobacillus* species in future NE studies, although it is currently unknown why they varied in their response to NE. Varying sensitivities among different *Lactobacillus* species perhaps help explain why they were unaltered by NE in some studies particularly when NE was relatively mild (Bortoluzzi et al., 2019; Kiu et al., 2019; Lu et al., 2020). Lactobacilli may not be unchanged in the jejunum or ileum if it is dominated by NE-resistant group A *Lactobacillus* and *L. reuteri*. It would also occur in the cecum where lactobacilli become minor. In agreement with this study, *L. johnsonii*, a NE-sensitive group B *Lactobacillus*, was reduced in the cecum of chicken in response to NE (Stanley et al., 2012). However, it remains unknown why *L. crispatus*, a group A *Lactobacillus*, was increased in NE chickens in the same study (Stanley et al., 2012), or the entire *Lactobacillus* population was increased by NE in several other studies (Lin et al., 2017; Latorre et al., 2018; Xu et al., 2018).

### Differential Sensitivity of Minor Ileal Bacteria to NE

Besides a reciprocal change in the relative abundance of the two most dominant bacteria in the ileum, a majority of less abundant bacteria were dramatically reduced as NE severity was increased. As a result, bacterial richness and Shannon diversity were precipitously reduced in severe NE, which is in agreement with earlier studies (Stanley et al., 2014; Yang et al., 2019). However, if an NE infection was mild, it is less likely to observe a significant difference in richness or Shannon Index between healthy and diseased ilea (Latorre et al., 2018; Hernandez-Patlan et al., 2019). It is also true that the cecal or fecal microbiota are less likely to be perturbed by NE than those in the small intestine (Li et al., 2017; Bortoluzzi et al., 2019; Yang et al., 2019) due to the fact that the microbiota in the lower GI tract are more diverse and thus more resilient to perturbations. Furthermore, the damages incurred by NE infection are generally limited to the upper GI tract while leaving the lower GI tract minimally affected.

**Figure 8:**
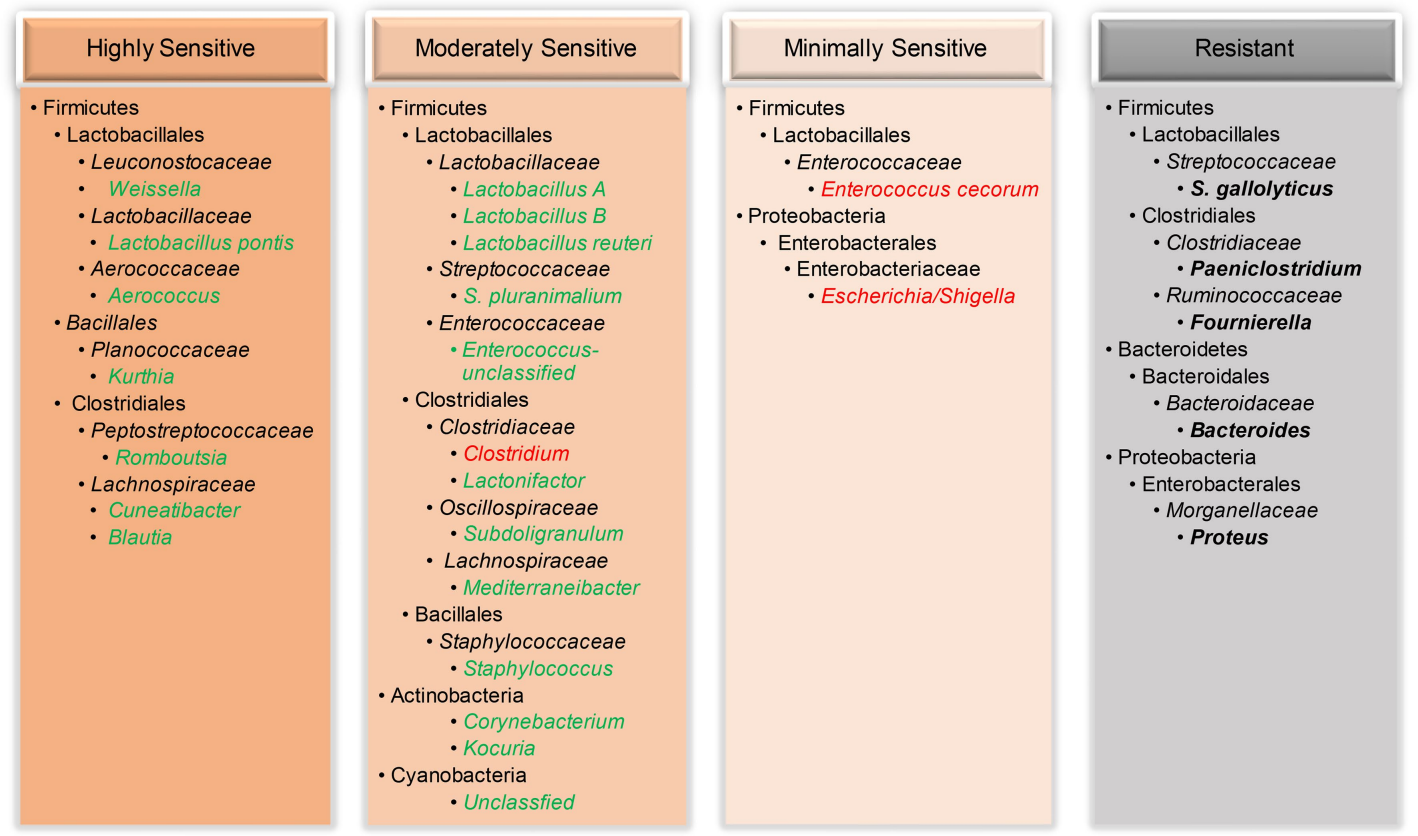
Classification of the ileal bacteria and their taxonomies based on their sensitivity to NE. While a majority of bacteria were abruptly vanished (highly sensitive) or gradually diminished (moderately sensitive) by NE, a few bacteria were only enriched during severe disease (minimally sensitive) or remained unchanged (resistant) by NE. Reduced bacterial taxa were indicated in green, and enriched bacteria were highlighted in red, while unaltered bacteria were indicated in bold black.

In this study, among all minor bacterial species in the ileum, some were highly sensitive to NE and largely abolished even during mild NE, while others were moderately sensitive showing a disease severity-dependent reduction (Figure 8). For example, two *Weissella* species (*W. paramesenteroides* and *W. thailandensis*) in the healthy ileum quickly vanished when encountering NE, consistent with earlier studies (Stanley et al., 2012; Lacey et al., 2018; Kiu et al., 2019; Yang et al., 2019). Interestingly, two related Cyanobacteria were also progressively declined in response to NE, which is in agreement with an earlier report (Zhang et al., 2018). While most bacteria in the ileum declined in response to NE, a small number of bacteria were minimally sensitive or rather resistant to colonization by *C. perfringens* (Figure 8). *Enterococcus cecorum* and two related *Escherichia*/*Shigella* species were unaltered by mild NE and even enriched in chickens with extremely severe NE, while bacteria such as *S. gallolyticus* remained largely unchanged by NE. An enrichment in acid-sensitive *Escherichia*/*Shigella* (Li et al., 2017; Yang et al., 2019) and *Enterococcus* (Hernandez-Patlan et al., 2019; Kiu et al., 2019) has also been reported in the cecum of NE-afflicted broilers and is likely due to a drastic diminishment of lactic acid-producing lactobacilli and a rise in pH in the ileum of severely infected chickens. An enrichment of *E. coli* could potentially induce local and systemic infections in chickens (Fancher et al., 2020), while *Shigella* may cause shigellosis with symptoms of diarrhea and bloody dysentery (Shi et al., 2014). Although *E. cecorum* is a commensal bacterium in the GI tract of mammals and poultry; it may cause enterococcal spondylitis in broilers (Jung et al., 2018). Therefore, if NE is left untreated, additional health complications to chickens may occur.

Notably, most phylogenetically related bacteria responded to NE similarly. Multiple species of *Blautia*, *Mediterraneibacter*, *Corynebacterium*, *Weissella*, and Cyanobacteria displayed a similar response to NE within each genus. However, intra-genus variations did exist. Besides four species of *Lactobacillus* showing an obvious differential response to NE, two *Streptococcus* species (*S. gallolyticus* F7 and *S. pluranimalium* F31), two *Enterococcus* species (F10 and F24), and another two *Subdoligranulum* species (F14 and F45) also responded to NE differently. These species-level variations within a genus have not been reported by earlier studies, highlighting a need for distinguishing among ASVs in future studies.

It is important to note that, although we could not pinpoint specific contributions of *E. maxima* and *C. perfringens* to the drastic shift of the ileal microbiota observed in this study, the outcome is most likely attributed to a combined effect of both, as either pathogen is incapable of eliciting severe intestinal pathologies and only leads to subtler and less obvious changes in the intestinal microbiota (Lin et al., 2017; Xu et al., 2018; Yang et al., 2019; Lu et al., 2020). It is also critical to understand the kinetic response of different intestinal microbes to NE in different segments of the intestinal tract to help understand the interactions and influences of these bacteria among each other in the context of NE.

### Functional Alterations of the Ileal Microbiota in Response to NE

Based on the PICRUSt prediction of the difference in functional potential of the ileal microbiota between mildly and severely infected chickens, we suggested that metabolisms of cofactors and vitamins, such as riboflavin, folate, thiamine, Vitamin B6, biotin, lipoic acid, and nicotinamide may be enhanced in severe NE. In agreement with our findings, biosynthesis of vitamins such as riboflavin biosynthesis pathways was also found to be suppressed in Crohn’s disease (Vich Vila et al., 2018). Additionally, the microbiota of chickens with severe NE had stronger overall amino acid metabolism than mild NE. We predicted that the biosynthesis of three aromatic amino acids, including tryptophan, tyrosine, and phenylalanine, as well as branched-chain amino acids, including valine, leucine, and isoleucine, was obviously increased in severely infected chickens. Given the fact that *C. perfringens* is predominant in severely infected ileum and lacks many genes involved in the biosynthesis of amino acids (Shimizu et al., 2002), amino acid synthetic pathways in the remaining bacterial population might be activated to compensate for an inability of *C. perfringens* to synthesize amino acids and support the colonization of *C. perfringens* in the ileum of NE chickens. Moreover, we predicted metabolic pathways of phenylalanine, valine, leucine, isoleucine, glycine, serine, and threonine were activated in severe NE, suggesting the capability of *C. perfringens* to utilize amino acids from the host or other microbes in the intestine to support its growth. In fact, growing demand for amino acids during NE is also consistent with enhanced NE susceptibility of chickens fed high protein diets (Moore, 2016).

One of the most striking findings was a clear reduction in the capacity for biosynthesis of secondary bile acids in severe NE. Secondary bile acids possess anti-inflammatory properties and are derived from primary bile acids by intestinal bacteria through deconjugation and dihydroxylation (Wahlstrom et al., 2016; Winston and Theriot, 2020). Biosynthesis of deoxycholic acid and lithocholic acid has been reported to be suppressed in human ulcerative colitis and their supplementation ameliorates inflammation and colitis in murine models (Sinha et al., 2020). Supplementation of deoxycholic acid, a secondary bile acid, has been shown to confer protection of chickens to NE (Wang et al., 2019a; Bansal et al., 2020). Sphingolipids are important immunomodulatory signaling molecules (Bryan et al., 2016). The sphingolipid metabolism pathway was also predicted to be deficient in severe NE, implying a possible link between sphingolipid and chicken NE. Consistently, decreased bacterial sphingolipids was observed in IBD patients (Brown et al., 2019). In addition, microbial fatty acid biosynthesis was enhanced in severe NE, which is in agreement with the fact that increased fatty acid synthesis contributes to intestinal inflammation as seen in IBD patients (Vich Vila et al., 2018).

It is noted that the above-mentioned functional potential changes are all predicted by PICRUSt2. Follow-up metagenomics, metatranscriptomics, and metametabolomics of the microbiota in the small and large intestines will help confirm the microbiota functions predicted in the current study and warrant further investigation.

### Manipulation of the Intestinal Microbiota for the Control and Prevention of NE

During the course of NE, the onset and severity of the disease were dictated by constant interaction and competition between *C. perfringens* and commensal intestinal microbiota. It is, therefore, critically important to understand whether the microbiota of the host affects *C. perfringens* colonization and intestinal pathology or *vice versa* (Antonissen et al., 2016). Consistent with an earlier study (Lacey et al., 2018), the intestinal microbiota of individual animals were relatively homogenous, but become extremely diverse in response to NE even for those animals displaying similar disease severities, suggesting that the diversity of the pre-existing microbiota is unlikely a major factor in NE development and that NE-induced microbiota changes are largely the consequence of *C. perfringens* colonization and intestinal inflammation. However, strategies to delay or alter the microbiota shifts may still prove beneficial in NE control and prevention by directly supplementing those diminished bacteria or indirectly cross-feeding them through prebiotics or probiotics. However, different microbes showed different sensitivities to NE. It is, therefore, interesting to directly compare the relative efficacy of different bacteria such as different species of *Lactobacillus* in NE control and prevention. Consistently, feeding various probiotics, such as *Lactobacillus*, *Enterococcus*, *Bacillus*, or yeast strains or prebiotics such as mannan-oligosaccharides or yeast extracts has shown a positive effect on the mitigation of NE (Caly et al., 2015; Fancher et al., 2020). Besides *Lactobacillus*, *Weissella* is another genus of lactic acid bacterium that is suppressed by NE. With the ability to produce bacteriocins and exopolysaccharides, *Weissella* is a commensal bacterium with beneficial antibacterial, anti-inflammatory, and antioxidative activities and has been explored as a potential probiotic candidate for use in animal feed (Kavitake et al., 2020). It will be interesting to evaluate the potential of *Weissella* against NE.

One significant discovery of this research was that a majority of bacteria that were diminished or disappeared in response to NE were short-chain fatty acid (SCFA)-producing clostridial bacteria, such as *Blautia*, *Mediterraneibacter*, *Romboutsia*, and *Subdoligranulum* in the families of *Lachnospiraceae*, *Peptostreptococcaceae*, and *Oscillospiraceae*. SCFAs are produced by bacterial fermentation of dietary fibers, contributing to host health by providing energy to epithelial cells and improving epithelial integrity and immune defense (Koh et al., 2016). Reduced production of SCFAs has been observed in human IBD patients (Vich Vila et al., 2018). In chickens, one earlier study has revealed a reduction in total SCFA concentrations in the ileum of NE-infected chickens, with acetate and propionate being reduced while butyrate increased (Li et al., 2017). Consistently, administration of SCFA-producing bacteria or SCFAs such as butyrate has shown a protective effect against NE (Timbermont et al., 2010; Jerzsele et al., 2012).

## Conclusion

In summary, we demonstrated for the first time a microbiota signature of disease severity-dependent reduction of a majority of commensal bacteria in the ileum of chickens in response to NE. Following a rapid expansion of *C. perfringens*, the ileal microbiota displayed more pronounced dysbiosis, which was mainly characterized by a progressive decline of lactic acid bacteria such as *Lactobacillus* and *Weissella* species and SCFA-producing bacteria, such as *Subdoligranulum*, *Blautia*, *Mediterraneibacter*, and *Romboutsia*. Interestingly, certain bacteria were more sensitive than others in response to NE infection. Most phylogenetically related bacteria similarly respond to NE, although intra-genus variations existed in several genera. Functionally, secondary bile acid biosynthesis may be suppressed by NE, while biosynthesis of aromatic and branched-amino acids may be enhanced in the ileum of NE-afflicted chickens. Our study unveiled shifts in intestinal microbiota in response to varying severities of chicken NE, which provides important leads for the future development of novel microbiota-based diagnostic or therapeutic approaches for the control and prevention of NE in poultry. A detailed illustration of structural and functional alterations of the intestinal microbiota of NE-infected chickens will provide opportunities to mitigate NE and possibly other enteric diseases in poultry.

## Data Availability Statement

The raw sequencing reads of this study have been deposited in Sequence Read Archive (SRA) at NCBI under the accession number BioProject PRJNA649841.

## Ethics Statement

The animal study was reviewed and approved by Institutional Animal Care and Use Committee of Oklahoma State University.

## Author Contributions

QY, JL, MW, SS, and KR conducted the animal trial and processed all samples. XW and JZ sequenced the ileal DNA samples on MiSeq. QY, JL, KR, XW, and JZ performed data analysis. QY drafted the manuscript. GZ conceived the study and revised the manuscript. All authors contributed to the article and approved the submitted version.

## Conflict of Interest

The authors declare that the research was conducted in the absence of any commercial or financial relationships that could be construed as a potential conflict of interest.

## Publisher’s Note

All claims expressed in this article are solely those of the authors and do not necessarily represent those of their affiliated organizations, or those of the publisher, the editors and the reviewers. Any product that may be evaluated in this article, or claim that may be made by its manufacturer, is not guaranteed or endorsed by the publisher.
