# Supplementary material for: Identification of an Intestinal Microbiota Signature Associated With the Severity of Necrotic Enteritis
Source: Front Microbiol. 2021 Aug 19;12:703693. doi: 10.3389/fmicb.2021.703693 (PMC8418326; doi:10.3389/fmicb.2021.703693)
Supplement: Supplementary file 1 [file Data_Sheet_1.docx]

Supplementary Material

# Supplemental Figures and Tables

**Figure S1.** Alpha and beta diversities of the ileal microbiota among chickens with varying severities of necrotic enteritis (NE). A group of 90 male Cobb broilers were subjected to NE, while another 10 chickens were mock-infected. Animals were separated into five groups according to their intestinal lesion scores. (**A**) Pielou's Evenness Index of the ileal microbiome was estimated and visualized using box and whisker plots. Each box indicates median, 25th and 75th percentiles, while whiskers extend to 1.5 interquartile range. Significance was measured using Kruskal-Wallis test, and pairwise comparisons were implemented using Wilcoxon rank sum test. Different superscripts denote significance (*P <* 0.05) in pairwise comparisons. (**B**) Principal coordinates analysis (PCoA) plots of unweighted UniFrac distances. Significance was determined using PERMANOVA.

**Figure S2.** Differential enrichment of the ileal bacteria between healthy chickens and chickens with mild NE. LEfSe analysis was performed to identify differentially abundant bacteria at the family (**A**), genus (**B**), and amplicon sequence variant (ASV) (**C**) levels between score-0 (healthy controls) and score-1 (mild NE) chickens with cut-offs *P* < 0.05 and LDA score > 2.0. (**D**) Phylogenetic relationships of differentially enriched bacteria. Differentially enriched bacterial taxa were represented by rings with the phylum shown in the outermost ring and the species in the innermost ring.

| **** | **Figure S3.** Differential enrichment of predicted microbial metabolic pathways between mild and severe NE. Functional potentials of ileal microbiota of score-1 (mild NE) and score-6 (severe NE) chickens were predicted using PICRUSt2, and differentially abundant level-3 KEGG pathways were determined in STAMP. Each bar represents a mean proportion (%) and SEM of a predicted KEGG pathway. The 95% confidence intervals of the differences in mean proportions between score-1 and score-6 chickens were also shown. Significance was determined by two-sided White’s non-parametric t-test, corrected with the Benjamini-Hochberg procedure, and indicated as q-values on the right. Only those pathways with q < 0.05 were displayed. |
| --- | --- |

**Table S1.** Pairwise comparisons of beta diversity of ileal microbiota of chickens

| Lesion Score | 0 | 1 | 2 | 5 | 6 |
| --- | --- | --- | --- | --- | --- |
| 0 |  | 0.007  (0.155) | 0.009  (0.202) | 0.001  (0.879) | 0.001  (0.717) |
| 1 | 0.001  (0.157) |  | 0.474  (0.025) | 0.002  (0.573) | 0.001  (0.600) |
| 2 | 0.002  (0.210) | 0.154  (0.049) |  | 0.001  (0.462) | 0.001  (0.546) |
| 5 | 0.001  (0.452) | 0.005  (0.192) | 0.113  (0.084) |  | 0.033  (0.170) |
| 6 | 0.001  (0.526) | 0.001  (0.350) | 0.001  (0.227) | 0.047  (0.124) |  |

**Note:** *P*-values and R^2^ (in parentheses) of pairwise comparisons of unweighted (shaded) and weighted UniFrac distances of the ileal microbiota in chickens with NE were determined by PERMANOVA using 999 permutations.

**Table S2**. Phylogenetic classification of bacteria using EzBioCloud

| Bacterial Feature | Top-hit taxon | Accession No. | Identity (%) | Top-hit taxonomy |
| --- | --- | --- | --- | --- |
| Lactobacillus A_F1 | *Lactobacillus crispatus, L. acidophilus, L. gallinarum* | AZCW01000112, CBLQ010000054, BALB01000057 | 100.00 | Bacteria;Firmicutes;Bacilli;Lactobacillales;Lactobacillaceae;Lactobacillus |
| Clostridium perfringens_F2 | *Clostridium perfringens* | CP000246 | 100.00 | Bacteria;Firmicutes;Clostridia;Clostridiales;Clostridiaceae;Clostridium |
| Lactobacillus B_F3 | *Lactobacillus johnsonii, L. gasseri* | ACGR01000047, CP000413 | 100.00 | Bacteria;Firmicutes;Bacilli;Lactobacillales;Lactobacillaceae;Lactobacillus |
| Lactobacillus reuteri_F4 | *Lactobacillus reuteri* | AP007281 | 100.00 | Bacteria;Firmicutes;Bacilli;Lactobacillales;Lactobacillaceae;Lactobacillus |
| Escherichia/Shigella_F5 | *Shigella flexneri,, Escherichia fergusonii* | X96963, CU928158 | 100.00 | Bacteria;Proteobacteria;Gammaproteobacteria;Enterobacterales;Enterobacteriaceae;Escherichia |
| Bacteroides fragilis_F6 | *Bacteroides fragilis* | CR626927 | 100.00 | Bacteria;Bacteroidetes;Bacteroidia;Bacteroidales;Bacteroidaceae;Bacteroides |
| Streptococcus gallolyticus_F7 | *Streptococcus gallolyticus* | FOLZ01000015 | 100.00 | Bacteria;Firmicutes;Bacilli;Lactobacillales;Streptococcaceae;Streptococcus;Streptococcus gallolyticus |
| Lactobacillus pontis_F8 | *Lactobacillus pontis* | AJ422032 | 100.00 | Bacteria;Firmicutes;Bacilli;Lactobacillales;Lactobacillaceae;Lactobacillus |
| Romboutsia_timonensis_F9 | *Romboutsia timonensis* | LT629862 | 100.00 | Bacteria;Firmicutes;Clostridia;Clostridiales;Peptostreptococcaceae;Romboutsia |
| Enterococcus cecorum_F10 | *Enterococcus cecorum* | ASWI01000003 | 99.60 | Bacteria;Firmicutes;Bacilli;Lactobacillales;Enterococcaceae;Enterococcus |
| Cyanobacteria_F11 | *HQ189092_s* | HQ189092 | 86.90 | Bacteria;Cyanobacteria;Chroobacteria;Pleurocapsales;Chroococcidiopsis_f;HQ189092_g |
| Kurthia populi_F12 | *Kurthia populi* | KM613132 | 100.00 | Bacteria;Firmicutes;Bacilli;Bacillales;Planococcaceae;Kurthia |
| Cyanobacteria_F13 | *HQ189092_s* | HQ189092 | 86.51 | Bacteria;Cyanobacteria;Chroobacteria;Pleurocapsales;Chroococcidiopsis_f;HQ189092_g |
| Subdoligranulum variabile_F14 | *Subdoligranulum variabile* | ACBY01000115 | 100.00 | Bacteria;Firmicutes;Clostridia;Clostridiales;Oscillospiraceae;Subdoligranulum |
| Proteus_F15 | *Proteus mirabilis, Cosenzaea myxofaciens* | ACLE01000013,  LXEN01000172 | 100.00  100.00 | Bacteria;Proteobacteria;Gammaproteobacteria;Enterobacterales;Morganellaceae;Proteus  Bacteria;Proteobacteria;Gammaproteobacteria;Enterobacterales;Morganellaceae;Cosenzaea |
| Fournierella massiliensis_F16 | *Fournierella massiliensis* | LN908959 | 100.00 | Bacteria;Firmicutes;Clostridia;Clostridiales;Oscillospiraceae;Fournierella |
| Weissella paramesenteroides_F17 | *Weissella paramesenteroides* | ACKU01000017 | 100.00 | Bacteria;Firmicutes;Bacilli;Lactobacillales;Leuconostocaceae;Weissella |
| Paeniclostridium sordellii_F18 | *Paeniclostridium sordellii* | AB075771 | 100.00 | Bacteria;Firmicutes;Clostridia;Clostridiales;Peptostreptococcaceae;Paraclostridium |
| Lactobacillus pontis_F19 | *Lactobacillus pontis* | AJ422032 | 99.60 | Bacteria;Firmicutes;Bacilli;Lactobacillales;Lactobacillaceae;Lactobacillus |
| Staphylococcus_F20 | *Staphylococcus saprophyticus, Staphylococcus xylosus, Staphylococcus cohnii* | AP008934, MRZO01000018, D83361 | 100.00 | Bacteria;Firmicutes;Bacilli;Bacillales;Staphylococcaceae;Staphylococcus |
| Cuneatibacter_F22 | *FCNS_s* | FCNS01000041 | 100.00 | Bacteria;Firmicutes;Clostridia;Clostridiales;Lachnospiraceae;Cuneatibacter |
| Blautia_F23 | *DQ456377_s* | DQ456377 | 100.00 | Bacteria;Firmicutes;Clostridia;Clostridiales;Lachnospiraceae;Blautia |
| Enterococcus_F24 | *Enterococcus faecalis, E. faecium, E. durans* | *ASDA01000001, AJ301830, BCQB01000108* | 100.00 | Bacteria;Firmicutes;Bacilli;Lactobacillales;Enterococcaceae;Enterococcus |
| Corynebacterium_F25 | *Corynebacterium glutamicum,*  *Corynebacterium efficiens,*  *Corynebacterium faecale* | BA000036, BA000035,  KT216265 | 99.60 | Bacteria;Actinobacteria;Actinomycetia;Mycobacteriales;Corynebacteriaceae;Corynebacterium |
| Corynebacterium_F28 | *Corynebacterium glutamicum,*  *Corynebacterium efficiens* | BA000036,  BA000035 | 100.00 | Bacteria;Actinobacteria;Actinomycetia;Mycobacteriales;Corynebacteriaceae;Corynebacterium |
| Blautia_F30 | *PAC002173_s* | PAC002173 | 99.60 | Bacteria;Firmicutes;Clostridia;Clostridiales;Lachnospiraceae;Blautia |
| Streptococcus pluranimalium_ F31 | *Streptococcus pluranimalium* | Y18026 | 100.00 | Bacteria;Firmicutes;Bacilli;Lactobacillales;Streptococcaceae;Streptococcus |
| Mediterraneibacter_F32 | *AJ315979_s* | AJ315979 | 97.61 | Bacteria;Firmicutes;Clostridia;Clostridiales;Lachnospiraceae;Mediterraneibacter |
| Kocuria_F33 | *Kocuria carniphila, Kocuria gwangalliensis, Kocuria atrinae* | AJ622907, EU286964, FJ607311 | 100.00 | Bacteria;Actinobacteria;Actinomycetia;Micrococcales;Micrococcaceae;Kocuria |
| Weissella thailandensis_F34 | *Weissella thailandensis* | AB023838 | 99.20 | Bacteria;Firmicutes;Bacilli;Lactobacillales;Leuconostocaceae;Weissella |
| Corynebacterium stationis_F37 | *Corynebacterium stationis* | CP009251 | 100.00 | Bacteria;Actinobacteria;Actinomycetia;Mycobacteriales;Corynebacteriaceae;Corynebacterium |
| Mediterraneibacter_F39 | *NFHL_s* | NFHL01000003 | 99.60 | Bacteria;Firmicutes;Clostridia;Clostridiales;Lachnospiraceae;Mediterraneibacter |
| Aerococcus_F41 | *Aerococcus viridans, Aerococcus urinaeequi* | ADNT01000041,CP014162 | 100.00 | Bacteria;Firmicutes;Bacilli;Lactobacillales;Aerococcaceae;Aerococcus |
| Mediterraneibacter_F43 | *DQ071451_s* | DQ071451 | 98.80 | Bacteria;Firmicutes;Clostridia;Clostridiales;Lachnospiraceae;Mediterraneibacter |
| Mediterraneibacter_F44 | *NFHL_s, LT635549_s* | NFHL01000003, LT635549 | 100.00 | Bacteria;Firmicutes;Clostridia;Clostridiales;Lachnospiraceae;Mediterraneibacter |
| Subdoligranulum_F45 | *DQ057482_s* | DQ057482 | 98.02 | Bacteria;Firmicutes;Clostridia;Clostridiales;Oscillospiraceae;Subdoligranulum |
| Lactonifactor_F47 | *DQ342330_s* | DQ342330 | 99.60 | Bacteria;Firmicutes;Clostridia;Clostridiales;Lachnospiraceae;Lactonifactor |
| Mediterraneibacter_F48 | *NFHL_s* | An76 | 99.21 | Bacteria;Firmicutes;Clostridia;Clostridiales;Lachnospiraceae;Mediterraneibacter |
| Sellimonas intestinalis F49 | *Sellimonas intestinalis* | LGAJ01000018 | 97.22 | Bacteria;Firmicutes;Clostridia;Clostridiales;Lachnospiraceae;Sellimonas |
| Lachnospiraceae_F50 | *DQ057459_s* | DQ057459 | 100.00 | Bacteria;Firmicutes;Clostridia;Clostridiales;Lachnospiraceae;DQ057459_g |
| Blautia_obeum_F51 | *Blautia obeum* | AAVO02000004 | 98.02 | Bacteria;Firmicutes;Clostridia;Clostridiales;Lachnospiraceae;Blautia |
| Anaerostipes butyraticus_F52 | *Anaerostipes butyraticus* | FJ947528 | 99.60 | Bacteria;Firmicutes;Clostridia;Clostridiales;Lachnospiraceae;Anaerostipes |
| Anaerobutyricum_F53 | *NFLV_s* | NFLV01000033 | 100.00 | Bacteria;Firmicutes;Clostridia;Clostridiales;Lachnospiraceae;Anaerobutyricum |
| Blautia_F54 | *NFJL_s* | NFJL01000055 | 100.00 | Bacteria;Firmicutes;Clostridia;Clostridiales;Lachnospiraceae;Blautia |
| Clostridium spiroforme_F55 | *Clostridium spiroforme* | X73441 | 100.00 | Bacteria;Firmicutes;Erysipelotrichia;Erysipelotrichales;Erysipelotrichaceae;Erysipelatoclostridium |
| Corynebacterium_F57 | *Corynebacterium xerosis, Corynebacterium freneyi* | LAYS01000008, AJ292762 | 100.00 | Bacteria;Actinobacteria;Actinomycetia;Mycobacteriales;Corynebacteriaceae;Corynebacterium |
| Rothia terrae_F60 | *Rothia terrae* | DQ822568 | 100.00 | Bacteria;Actinobacteria;Actinomycetia;Micrococcales;Micrococcaceae;Rothia |
| Blautia_F61 | *DQ456377_s* | DQ456377 | 96.43 | Bacteria;Firmicutes;Clostridia;Clostridiales;Lachnospiraceae;Blautia |
| Mediterraneibacter_F62 | *Ruminococcus lactaris* | ABOU02000049 | 96.83 | Bacteria;Firmicutes;Clostridia;Clostridiales;Lachnospiraceae;Mediterraneibacter |
| Blautia_F63 | *DQ057459_s* | DQ456377 | 96.03 | Bacteria;Firmicutes;Clostridia;Clostridiales;Lachnospiraceae;Blautia |
| Merdimonas faecis_F64 | *Merdimonas faecis* | KP966093 | 100.00 | Bacteria;Firmicutes;Clostridia;Clostridiales;Lachnospiraceae;Merdimonas |
| Lachnospiraceae_F65 | *Kineothrix alysoides* | JPNB01000002 | 96.03 | Bacteria;Firmicutes;Clostridia;Clostridiales;Lachnospiraceae;Kineothrix |
| Massiliomicrobiota timonensis_F66 | *Massilimicrobiota timonensis* | UYXN01000093 | 100.00 | Bacteria;Firmicutes;Tissierellia;Tissierellales;Peptoniphilaceae;Massilimicrobiota |
| Escherichia_F68 | *Shigella dysenteriae* | X96966 | 95.24 | Bacteria;Proteobacteria;Gammaproteobacteria;Enterobacterales;Enterobacteriaceae;Escherichia |
| Blautia_F72 | *DQ057417_s* | DQ057417 | 99.60 | Bacteria;Firmicutes;Clostridia;Clostridiales;Lachnospiraceae;Blautia |
| Facklamia tabacinasalis_F76 | *Facklamia tabacinasalis* | Y17820 | 100.00 | Bacteria;Firmicutes;Bacilli;Lactobacillales;Aerococcaceae;Facklamia |
| Anaerobutyricum_F79 | *DQ793914_s* | DQ793914 | 100.00 | Bacteria;Firmicutes;Clostridia;Clostridiales;Lachnospiraceae;Anaerobutyricum |
| Corynebacterium_F80 | *Corynebacterium testudinoris, Corynebacterium atrinae* | CP011545, KF135179 | 100.00 | Bacteria;Actinobacteria;Actinomycetia;Mycobacteriales;Corynebacteriaceae;Corynebacterium |
| Mediterraneibacter_F81 | *EF025265_s* | EF025265 | 100.00 | Bacteria;Firmicutes;Clostridia;Clostridiales;Lachnospiraceae;Mediterraneibacter |
| Lactonifactor _F88 | *DQ342330_s* | DQ342330 | 100.00 | Bacteria;Firmicutes;Clostridia;Clostridiales;Lachnospiraceae;Lactonifactor |
| Corynebacterium sputi_F89 | *Corynebacterium sputi* | AM930556 | 100.00 | Bacteria;Actinobacteria;Actinomycetia;Mycobacteriales;Corynebacteriaceae;Corynebacterium |
| Ochrobactrum pseudogrignonense_F92 | *Ochrobactrum pseudogrignonense* | NNRM01000012 | 100.00 | Bacteria;Proteobacteria;Alphaproteobacteria;Rhizobiales;Brucellaceae;Ochrobactrum |
| Sellimonas_F94 | *Sellimonas intestinalis* | LGAJ01000018 | 95.63 | Bacteria;Firmicutes;Clostridia;Clostridiales;Lachnospiraceae;Sellimonas |
| Oscillospiraceae_F95 | *PAC000672_s* | PAC000672 | 95.63 | Bacteria;Firmicutes;Clostridia;Clostridiales;Oscillospiraceae;PAC000672_g |
| Faecalibacterium_F98 | *DQ455832_s* | DQ455832 | 100.00 | Bacteria;Firmicutes;Clostridia;Clostridiales;Oscillospiraceae;Faecalibacterium |
| Lachnospiraceae_F103 | *PAC002721_s* | PAC002721 | 98.02 | Bacteria;Firmicutes;Clostridia;Clostridiales;Lachnospiraceae;PAC001043_g |
| Lachnoclostridium phocaeense_F125 | *Lachnoclostridium phocaeense* | LT635479 | 100.00 | Bacteria;Firmicutes;Clostridia;Clostridiales;Lachnospiraceae;Lachnoclostridium |
| Lachnospiraceae_F144 | *PAC002721_s* | PAC002721 | 97.62 | Bacteria;Firmicutes;Clostridia;Clostridiales;Lachnospiraceae;PAC001043_g |

**Note:** Bacterial taxonomies were classified according to the top hit identified by EzBioCloud (https://www.ezbiocloud.net/identify). If more than three hits showing 100% identity, only 2-3 representative species that are known to be present in the intestinal tract are shown for the sake of simplicity. If the top hit shows < 97% identity, the taxon is classified to one upper level. Phylogenetic classifications of top 20 bacterial amplicon sequence variants (ASVs) and representative NE-associated bacteria were displayed.

**Table S3**. Relative abundance (%) of the ileal bacterial phyla, families, genera, and ASVs in healthy and NE chickens

| Taxon | Score-0 | Score-1 | Score-2 | | Score-5 | Score-6 | | *P*-Value | FDR |  |
| --- | --- | --- | --- | --- | --- | --- | --- | --- | --- | --- |
| Phyla |  | | |  | | |  | | | |
| Firmicutes | 98.08 ± 0.59^ab^ | 95.28 ± 1.34^ac^ | 97.70 ± 0.81^a^ | | 99.39 ± 0.53^b^ | 83.59 ± 4.36^c^ | | 0.004 | 0.005 |  |
| Proteobacteria | 0.16 ± 0.04^a^ | 3.32 ± 1.42^a^ | 1.46 ± 0.75^a^ | | 0.54 ± 0.48^a^ | 13.01 ± 2 .96^b^ | | 0.002 | 0.003 |  |
| Bacteroidetes | 0.003 ± 0.002 | 0.002 + 0.001 | 0.02 ± 0.01 | | 0.001 ± 0.001 | 3.38 ± 1.91 | | 0.356 | 0.356 |  |
| Cyanobacteria | 0.99 ± 0.41^a^ | 0.94 ± 0.31^ab^ | 0.46 ± 0.20^bc^ | | 0.04 ± 0.04^cd^ | 0.003 ± 0.002^d^ | | 2.14E-05 | 5.35E-05 |  |
| Actinobacteria | 0.76 ± 0.20^a^ | 0.46 ± 0.14^ab^ | 0.36 ± 0.12^bc^ | | 0.03 ± 0.01^cd^ | 0.01 ± 0.01^d^ | | 1.69E-05 | 5.35E-05 |  |
| Families |  | | |  | | |  | | | |
| Lactobacillaceae | 91.00 ± 1.16^a^ | 79.98 ± 4.62^b^ | 75.30 ± 5.84^ab^ | | 29.06 ± 7.38^c^ | 19.07 ± 3.74^c^ | | 2.19E-07 | 1.10E-06 |  |
| Clostridiaceae | 0.21 ± 0.04^a^ | 12.65 ± 3.81^b^ | 20.51 ± 6.02^b^ | | 70.18 ± 7.58^c^ | 58.19 ± 3.95^c^ | | 2.19E-08 | 2.19E-07 |  |
| Enterobacteriaceae | 0.13 ± 0.04^a^ | 3.30 ± 1.42^a^ | 1.45 ± 0.75^a^ | | 0.54 ± 0.48^a^ | 12.93 ± 2.94^b^ | | 0.001 | 0.002 |  |
| Bacteroidaceae | 0.003 ± 0.002 | 0.002 ± 0.001 | 0.02 ± 0.01 | | 0.001 ± 0.001 | 3.38 ± 1.91 | | 0.356 | 0.356 |  |
| Streptococcaceae | 0.29 ± 0.06 | 0.93 ± 0.64 | 0.30 ± 0.16 | | 0.05 ± 0.02 | 4.73 ± 2.20 | | 0.120 | 0.133 |  |
| Lachnospiraceae | 2.49 ± 0.43^a^ | 0.54 ± 0.12^b^ | 0.42 ± 0.20^b^ | | 0.02 ± 0.02^c^ | 0.14 ± 0.12^c^ | | 3.51E-07 | 1.17E-06 |  |
| Cyanobacteria_unidentified | 0.99 ± 0.41^a^ | 0.94 ± 0.31^ab^ | 0.46 ± 0.20^bc^ | | 0.04 ± 0.04^cd^ | 0.003 ± 0.002^d^ | | 2.14E-07 | 4.28E-06 |  |
| Peptostreptococcaceae | 1.36 ± 0.62^a^ | 0.17 ± 0.14^b^ | 0.15 ± 0.11^bc^ | | 0.00^c^ | 0.03 ± 0.02^c^ | | 8.28E-06 | 2.07E-05 |  |
| Enterococcaceae | 0.34 ± 0.06^a^ | 0.24 ± 0.08^a^ | 0.40 ± 0.17^ac^ | | 0.03 ± 0.03^b^ | 1.23 ± 0.43^c^ | | 0.005 | 0.006 |  |
| Oscillospiraceae | 0.79 ± 0.15^a^ | 0.35 ± 0.06^b^ | 0.38 ± 0.17^bc^ | | 0.04 ± 0.02^cd^ | 0.19 ± 0.14^d^ | | 1.17E-05 | 1.94E-05 |  |
| Genera |  |  |  | |  |  | |  |  |  |
| Lactobacillus | 90.97 ± 1.16^a^ | 79.97 ± 4.62^b^ | 75.29 ± 5.83^ab^ | | 29.06 ± 7.38^c^ | 19.07 ± 3.74^c^ | | 2.19E-07 | 7.12E-07 |  |
| Clostridium | 0.02 ± 0.01^a^ | 12.58 ± 3.82^b^ | 20.47 ± 6.02^b^ | | 70.17 ± 7.58^c^ | 58.14 ± 3.97^c^ | | 1.07E-08 | 1.07E-07 |  |
| Escherichia/Shigella | 0.13 ± 0.04^a^ | 3.23 ± 1.40^a^ | 1.43 ± 0.74^a^ | | 0.54 ± 0.48^a^ | 12.92 ± 2.94^b^ | | 0.002 | 0.002 |  |
| Bacteroides | 0.003 ± 0.002 | 0.002 ± 0.001 | 0.02 ± 0.01 | | 0.002 ± 0.002 | 3.38 ± 1.91 | | 0.356 | 0.356 |  |
| Streptococcus | 0.27 ± 0.06 | 0.93 ± 0.64 | 0.30 ± 0.16 | | 0.05 ± 0.02 | 4.73 ± 2.20 | | 0.135 | 0.150 |  |
| Cyanobacteria_unidentified | 0.99 ± 0.41^a^ | 0.94 ± 0.31^ab^ | 0.46 ± 0.20^bc^ | | 0.04 ± 0.04^cd^ | 0.003 ± 0.002^d^ | | 2.14E-05 | 3.57E-05 |  |
| Romboutsia | 1.31 ± 0.63^a^ | 0.17 ± 0.14^b^ | 0.09 ± 0.06^bc^ | | 0.00^cd^ | 0.003 ± 0.003^d^ | | 3.63E-06 | 7.26E-06 |  |
| Enterococcus | 0.34 ± 0.06^ab^ | 0.24 ± 0.08^a^ | 0.40 ± 0.17^a^ | | 0.03 ± 0.03^c^ | 1.23 ± 0.43^b^ | | 0.005 | 0.006 |  |
| Kurthia | 0.62± 0.21^a^ | 0.004 ± 0.002^b^ | 0.003 ± 0.002^b^ | | 0.00^b^ | 0.001 ± 0.001^b^ | | 4.28E-08 | 2.14E-07 |  |
| Subdoligranulum | 0.61 ± 0.11^a^ | 0.31 ± 0.06^b^ | 0.34 ± 0.16^b^ | | 0.03 ± 0.02^c^ | 0.004 ± 0.002^c^ | | 2.85E-07 | 7.12E-07 |  |
| ASVs |  |  |  | |  |  | |  |  |  |
| Lactobacillus A_F1 | 57.12 ± 3.03^a^ | 59.10 ± 4.41^a^ | 54.14 ± 4.42^a^ | | 20.07 ± 6.31^b^ | 13.11 ± 3.17^b^ | | 1.01E-06 | 4.56E-06 |  |
| Clostridium perfringens_F2 | 0.02 ± 0.01^a^ | 12.54 ± 3.83^b^ | 20.46 ± 6.02^b^ | | 70.17 ± 7.58^c^ | 58.14 ± 3.97^c^ | | 1.07E-08 | 2.13E-07 |  |
| Lactobacillus B_F3 | 20.76 ± 3.11^a^ | 10.99 ± 1.77^b^ | 10.90 ± 2.30^b^ | | 2.89 ± 1.24^c^ | 2.14 ± 0.56^c^ | | 8.49E-07 | 4.56E-06 |  |
| Lactobacillus reuteri_F4 | 10.73 ± 0.67^a^ | 9.32 ± 1.34^ab^ | 9.50 ± 1.44^ab^ | | 5.72 ± 1.66^bc^ | 2.91 ± 0.45^c^ | | 1.34E-04 | 2.45E-04 |  |
| Escherichia/Shigella_F5 | 0.13 ± 0.04^a^ | 3.23 ± 1.40^a^ | 1.42 ± 0.74^a^ | | 0.54 ± 0.48^a^ | 12.92 ± 2.94^b^ | | 0.002 | 0.003 |  |
| Bacteroides fragilis_F6 | 0.003 ± 0.002 | 0.002 ± 0.001 | 0.02 ± 0.01 | | 0.001 ± 0.001 | 3.38 ± 1.91 | | 0.356 | 0.418 |  |
| Streptococcus gallolyticus_F7 | 0.06 ± 0.03 | 0.87 ± 0.64 | 0.24 ± 0.16 | | 0.04 ± 0.02 | 4.73 ± 2.20 | | 0.574 | 0.638 |  |
| Lactobacillus pontis_F8 | 2.10 ± 0.42^a^ | 0.52 ± 0.26^b^ | 0.66 ± 0.23^b^ | | 0.33 ± 0.33^b^ | 0.78 ± 0.28^b^ | | 0.002 | 0.004 |  |
| Romboutsia timonensis_F9 | 1.31 ± 0.63^a^ | 0.17 ± 0.14^b^ | 0.09 ± 0.06^bc^ | | 0.00^cd^ | 0.003 ± 0.003^d^ | | 3.63E-06 | 1.04E-05 |  |
| Enterococcus cecorum_F10 | 0.10 ± 0.02^a^ | 0.12 ± 0.04^a^ | 0.27 ± 0.15^ab^ | | 0.03 ± 0.03^b^ | 1.23 ± 0.43^c^ | | 0.004 | 0.005 |  |
| Cyanobacteria_F11 | 0.72 ± 0.32^a^ | 0.33 ± 0.09^ab^ | 0.29 ± 0.16^bc^ | | 0.02 ± 0.02^cd^ | 0.001 ± 0.001^d^ | | 2.14E-05 | 5.35E-05 |  |
| Kurthia populi_F12 | 0.50 ± 0.22^a^ | 0.002 ± 0.002^b^ | 0.00^b^ | | 0.00^b^ | 0.001 ± 0.001^b^ | | 1.14E-06 | 4.56E-06 |  |
| Cyanobacteria_F13 | 027 ± 0.10^a^ | 0.60 ± 0.25^a^ | 0.17 ± 0.06^a^ | | 0.02 ± 0.02^cb^ | 0.002 ± 0.002^b^ | | 5.78E-05 | 1.28E-04 |  |
| Subdoligranulum variabile_F14 | 0.53 ± 0.10^a^ | 0.30 ± 0.06^ab^ | 0.32 ± 0.15^b^ | | 0.03 ± 0.02^c^ | 0.002 ± 0.001^c^ | | 2.91E-07 | 2.91E-06 |  |
| Proteus_F15 | 0.00 | 0.003 ± 0.003 | 0.003 ± 0.002 | | 0.00 | 0.08 ± 0.07 | | 0.612 | 0.644 |  |
| Fournierella massiliensis_F16 | 0.002 ± 0.002 | 0.00 | 0.00 | | 0.00 | 0.05 ± 0.05 | | 0.274 | 0.342 |  |
| Weissella paramesenteroides_F17 | 0.22 ± 0.04^a^ | 0.18 ± 0.16^b^ | 0.08 ± 0..04^bc^ | | 0.005 ± 0.005^bc^ | 0.002 ± 0.001^c^ | | 6.71E-05 | 1.34E-04 |  |
| Paeniclostridium sordellii_F18 | 0.01 ± 0.01 | 0.002 ± 0.001 | 0.06 ± 0.06 | | 0.00 | 0.03 ± 0.02 | | 0.883 | 0.883 |  |
| Lactobacillus pontis_F19 | 0.26 ± 0.06^a^ | 0.04 ± 0.03^b^ | 0.09 ± 0.03^bc^ | | 0.02 ± 0.02^bc^ | 0.12 ± 0.05^ac^ | | 0.006 | 0.008 |  |
| Staphylococcus_F20 | 0.31 ± 0.06^a^ | 0.12 ± 0.03^b^ | 0.10 ± 0.03^b^ | | 0.002 ± 0.002^bc^ | 0.001 ± 0.001^c^ | | 1.49E-06 | 4.97E-06 |  |

**Note**: The relative abundances of (%) of top five phyla, top 10 families, top 10 genera, and top 20 ASVs of ileal microbiota in chickens with varied lesion scores were shown as means and SEM. Statistical significance was determined using the Kruskal-Wallis test, followed by the pairwise Wilcoxon rank sum test. Significance was further corrected with the Benjamini-Hochberg procedure and indicated as false discovery rate (FDR). FDR < 0.05 was considered significant.
